# Expression QTL (eQTLs) Analyses Reveal Candidate Genes Associated With Fruit Flesh Softening Rate in Peach [*Prunus persica* (L.) Batsch]

**DOI:** 10.3389/fpls.2019.01581

**Published:** 2019-12-03

**Authors:** Tomás Carrasco-Valenzuela, Claudia Muñoz-Espinoza, Aníbal Riveros, Romina Pedreschi, Pere Arús, Reinaldo Campos-Vargas, Claudio Meneses

**Affiliations:** ^1^Centro de Biotecnología Vegetal, Facultad de Ciencias de la Vida, Universidad Andres Bello, Santiago, Chile; ^2^Escuela de Agronomía, Pontificia Universidad Católica de Valparaíso, Quillota, Chile; ^3^IRTA, Centre de Recerca en Agrigenòmica (CSIC-IRTA-UAB-UB), Barcelona, Spain; ^4^FONDAP Center for Genome Regulation, Santiago, Chile

**Keywords:** expression quantitative trait loci, *Prunus persica*, softening rate, flesh firmness, RNA-seq

## Abstract

Significant differences in softening rate have been reported between melting flesh in peach and nectarine varieties. This trait seems to be controlled by several genes. We aimed to identify candidate genes involved in fruit softening rate by integrating quantitative trait loci (QTL) and expression QTL (eQTL) analyses, comparing siblings with contrasting softening rates. We used a segregating population derived from nectarine cv. ‘Venus’ selfing, which was phenotyped for softening rate during three seasons. Six siblings with high (HSR) and six with low softening rate (LSR) were sequenced using RNA-Seq. A group of 5,041 differentially expressed genes was identified. Also, we found a QTL with a LOD (logarithm of odds) score of 9.7 on LG4 in all analyzed seasons. Furthermore, we detected 1,062 eQTLs, of which 133 were found co-localizing with the identified QTL. Gene Ontology (GO) analysis showed ‘Response to auxin’ as one the main over-represented categories. Our findings suggest over-expression of auxin biosynthetic related genes in the HSR group, which implies a higher expression and/or accumulation of auxin, thereby triggering fast softening. Conversely, the LSR phenotype might be explained by an altered auxin-homeostasis associated with low auxin levels. This work will contribute to unraveling the genetic mechanisms responsible for the softening rate in peaches and nectarines and lead to the development of molecular markers.

## Introduction

Peach [*Prunus persica* (L.) Batsch] is the second most predominant temperate tree fruit species in worldwide production. In fact, peach production rose to 24.9 million tons of fruits in 2016, representing a cultivated area of 1,639,925 ha throughout the world (FAOSTAT, 2018). Peach is a self-compatible fruit species, diploid, and exhibits a relatively short juvenile period (2–3 years) (Arús et al., 2012).

Peach belongs to the Rosaceae family (Bassi and Monet 2008; Shulaev et al., 2008), and has become the most economically important crop in Prunus (Abbott et al., 2002), a genus that also includes nectarine, plum, apricot, cherry, and almond (Arús et al., 2012). In addition, peach is one of the best genetically characterized species in the Rosaceae family (Ogundiwin et al., 2009) and it has been used as a model for genetics and genomics studies of tree fruit species (Byrne, 1990; Abbott et al., 2002; Shulaev et al., 2008; Zhebentyayeva et al., 2008; Arús et al., 2012). Also, peach has a small genome (ca. 224.6 Mb) (Verde et al., 2013).

Furthermore, peach breeding programs require a relatively short time to obtain crossings and marketable products relative to other tree fruit crops, which is an advantage when developing new cultivars for dynamic markets (Infante et al., 2008). In fact, breeding efforts have been focused on the selection of traits associated with fruit appearance and textural attributes (Cantín et al., 2010), as well as quality fruit traits including flavor, aroma, texture, and nutritional attributes, such as fruit size, color, firmness, resistance to postharvest handling, and an extended shelf life (Cirilli et al., 2016). Thus, a better understanding of the genetic and physiological basis of fruit quality traits could pave the way for a more efficient new cultivar development process (Infante et al., 2008).

Fruit ripening encompasses several coordinated, genetically programmed events (biochemical and physiological). During ripening, the fruit texture changes, resulting in a decrease in firmness accompanied by an increase of ethylene release and up-regulated expression of genes, often triggered by plant hormones (Tonutti et al., 1991; Trainotti et al., 2007; Zhang et al., 2017). Also, changes in the composition and ratio of fruit inclusion, fruit color, sugar accumulation, aromatic compound production, and fruit softening are induced (Crisosto, 1994; Gapper et al., 2013; Zhang et al., 2017). Modifications and remodeling of the cell wall polysaccharide composition occur, triggering fruit softening (Hayama et al., 2006; Kumar et al., 2014).

Peach is a climacteric fruit. The peach fruit respiration rate undergoes a measurable peak before the onset of the ripening process, as well as an ethylene production peak (Trainotti et al., 2007). As a climacteric fruit, peach ripening is mainly controlled by ethylene (Liu et al., 2015). Concomitant with the ethylene production peak, auxin increases have also been detected in climacteric fruits (Trainotti et al., 2007; Liu et al., 2015). Also, it has been suggested that auxin has a role in ethylene biosynthesis and signaling genes expression regulation in peach and tomato (Trainotti et al., 2007; Pirrello et al., 2012; Liu et al., 2015). In fact, several reports have suggested a possible relationship between fruit softening and auxin at ripening (Miller et al., 1987; Agusti et al., 1999; Ohmiya, 2000; Tatsuki et al., 2013).

Considering that the sensory and nutritional characteristics defining fruit quality traits are determined at the ripening stage, the identification of genetic and molecular factors acting as key drivers of ripening will allow improvements to be made to the overall fruit quality (Carrari and Fernie, 2006; García-Gómez et al., 2019), as well as to preserve fruit quality attributes during the postharvest shelf life (Liu et al., 2015).

Flesh firmness is an important indicator of fruit quality and freshness for peach consumers (Infante et al., 2008). Hence, softening constitutes a key aspect determining peach fruit shelf life. As a climacteric fruit, peach softening occurs in two stages: first, fruit firmness decreases continuously and slowly; in the second stage, known as the ‘melting’ stage, the fruit firmness rapidly decreases (Lester et al., 1994; Ghiani et al., 2011).

Fruits are harvested at physiological maturity, and ripening happens off the tree. Thus, responsiveness to postharvest conditions represents a quality trait for climacteric fruits, such as peaches, significantly affecting softening (Ramina et al., 2008; Eduardo et al., 2011; Serra et al., 2017). Since the fruit is destined to distant markets, then quality traits such as firmer texture and very slow softening rates are the main targets of breeding programs, according to consumer preferences (Eduardo et al., 2011).

Considering fruit firmness and texture at the ripe stage, two main types have been characterized, both at the genetic and physiological level, known as non-melting flesh (NMF) and melting flesh (MF). NMF shows a very slow softening without a significant reduction in flesh firmness (Tatsuki et al., 2013), although red coloration is usually absent at ripening. However, NMF cultivars are mainly used for canning purposes, based on fruit integrity after heat processing (Ghiani et al., 2011). Conversely, MF exhibits a rapid softening rate after harvest, resulting in fruit with a short shelf life (Tatsuki et al., 2013). Nonetheless, most fresh market peaches belong to the MF group, showing a rapid softening at ripening (Infante et al., 2008; Ghiani et al., 2011).

Regarding fruit softening differences observed between MF and NMF peach cultivars, a sustained increase in soluble pectin is associated with the MF phenotype, which is associated with progressive pectin depolymerization. In contrast, in the NMF cultivars, fruits maintain firmness at ripening as a result of the scarce solubilization or depolymerization of pectin (Brummell et al., 2004; Ghiani et al., 2011).

It has been proposed that this is a result of the cell wall remodeling enzymes [e.g., endo-polygalacturonase (endoPG)] during ripening (Pressey and Avants, 1978; Morgutti et al., 2006). Evidence suggests that this trait is under the control of a major gene (*M*/*m*) localized on linkage group 4 (LG4) (Peace et al., 2005). In fact, in the position of the *M* locus, there are two genes coding for endoPG, one of which (*Prupe.4G261900*) has been proposed as a candidate for the determination of this trait (Lester et al., 1996; Peace et al., 2005; Morgutti et al., 2006).

Also, a high association between the pit/flesh adherence trait and flesh texture in peach – resulting in the combination of freestone (*F*) melting (*M*) peaches or clingstone (f) NMF (m) fruits – has been reported (Infante et al., 2008). Two other types have been recently reported, denominated ‘stony hard’ (Tatsuki et al., 2006; Pan et al., 2015) and ‘slow ripening’ (Brecht et al., 1982; Nuñez-Lillo et al., 2015). In the former, ethylene production and the subsequent softening in mature fruits are absent, possibly a result of a mutation in the ethylene biosynthesis pathway (Haji et al., 2001; Infante et al., 2008). In the latter, a mutation prevents the normal ripening process, which seems to be under control of a single gene (*S*r/*sr*). Individuals exhibiting this phenotype are often discarded from breeding programs (Meneses et al., 2017).

Differences in the softening rate occur have been detected among MF varieties. One of these, described as slow-melting flesh (SMF) trait, is characterized by a significant decrease in the postharvest softening rate, providing a longer shelf life and a delayed harvest-time, ultimately resulting in an improved fruit quality (Serra et al., 2017). Hence, SMF varieties have become standard in the peach industry due to their higher fruit quality and better handling after harvest (Laurens et al., 2018). However, the underlying genetic mechanisms controlling characters related to postharvest shelf life, including softening rate (SOR) and SMF, have only been partially elucidated (Serra et al., 2017).

Several studies have focused on peach in efforts to identify associations between major genes, QTLs, and candidate genes for fruit quality traits (Etienne et al., 2002; Infante et al., 2008; Eduardo et al., 2011; Fresnedo-Ramírez et al., 2015), as well as in other *Prunus* species, such as apricot (Salazar et al., 2014; García-Gómez et al., 2019) and Japanese plum (Salazar et al., 2017); for a review, see Aranzana et al. (2019).

The advent of Expression Quantitative Trait Loci (eQTLs) analysis has strengthened ongoing efforts focused on improving our understanding of how genetic variants affect gene expression (Rockman and Kruglyak, 2006; Cookson et al., 2009). Hence, eQTL analysis is based on the use of gene expressions as quantitative molecular phenotypes to identify genetic variants significantly associated with gene expression modifications (Stranger et al., 2007).

Recently, high-throughput Next Generation Sequencing technologies (NGS), including microarrays and RNA-Seq, have been applied to study genetic variation in gene expression, representing an important source for eQTL analysis. An advantage of this methodology is its suitability to studying the genetic architecture of complex traits (Ye et al., 2014; Galpaz et al., 2018).

Therefore, in order to determine candidate genes related to targeted traits, transcript abundances had been used as quantitative trait data in the QTL analysis (Druka et al., 2010; Galpaz et al., 2018; García-Gómez et al., 2019). Multiple authors have noted the usefulness of gene expression analysis based on qPCR for the experimental validation of QTLs in fruit crops (Huang et al., 2014; Sugiyama et al., 2014; García-Gómez et al., 2019), confirming the correlation between genotype and gene expression levels (Conesa et al., 2016).

The main aim of this work was to elucidate genetic factors associated with softening rate (SOR) during peach ripening. Second, using transcriptomic analyses siblings with contrasting softening rate phenotypes, we aim to identify QTLs and eQTLs. Third, we aim to integrate findings from both approaches to select candidate genes involved in fruit softening rate during peach maturation and to validate these by qPCR.

## Materials and Methods

### Plant Material

The mapping population (‘Venus’ × ‘Venus’; V×V; N = 138) was obtained from self-pollination of *Prunus persica* cv. Venus, a freestone nectarine with yellow melting flesh. This population is located at Rayentué Experimental Station of the Instituto de Investigaciones Agropecuarias (INIA), VI Region, Chile (34°24’S and 70°50’W). The mapping population V×V consists of 9-year-old trees in an experimental orchard, which were grafted over cv. ‘Nemaguard’, and planted at 1 × 3 m spacing. This population segregates for peach fruit quality traits, including mealiness, solid soluble content, titrable acidity, and maturity date, among others (Nuñez-Lillo et al., 2015). Siblings were managed under standard conditions for watering, fertilization, pest and diseases control, and pruning.

### Fruit Phenotyping

Individuals were sampled to evaluate fruit quality traits, such as soluble solids content (°Brix), titratable acidity (%), fruit weight (g), and firmness (Newtons, N). The total of siblings phenotypically evaluated per year varied from 104 to 113 plants, depending on plant vigor and fruit availability (**Supplementary Table 1**).

Peach mature fruits were harvest at the field in the morning and later transported to the laboratory. Fruits exhibiting optimum commercial maturity, considering fruit firmness values reached 50.0 ± 2.0 Newtons and chlorophyll absorbance at harvest (I_AD_) values between 0.8–1.5 (DA-Meter FR Turoni, Forlí, Italia) (Lurie et al., 2013), uniform size, and without defectives were selected for phenotypic evaluations. Evaluations were performed using nine fruits per sibling, and fruit background color was considered as the harvest index parameter (Nilo et al., 2012) (**Supplementary Table 1**).

Fruit firmness was evaluated on the two opposite cheeks of the fruit, after removing the fruit skin, using a fruit pressure tester with an 8 mm cylindrical plunger (Effigi, Alfonsine, Italia). Measurements were carried out at harvest and harvest plus 3 days at 20°C, under conventional atmosphere, and 12 hours of light and 8 h of dark, denominated as shelf life stage according with Serra et al. (2017) and Giné-Bordonaba et al. (2016). Also, the softening rate was estimated using five fruits per sibling, as the ratio between observed firmness at harvest and at shelf life, according to the following equation:

(1) Softening rate=100×[1−(Fm SL)/Fm HV]

Where Fm SL corresponds to the flesh firmness measurement observed at shelf life, while Fm HV represents the flesh firmness at harvest, and both are expressed as Newton (N).

### Experimental Design and Sample Collection

A group of 12 siblings of V×V with contrasting phenotypes for softening rate, *i.e.*, higher and lower softening rate, named as LSR-1 to LSR6 and HSR-1 to HSR6 were analyzed using ANOVA and the post-hoc Tukey test (**Supplementary Table 2**). Subsequently, these groups of individuals were selected to develop an RNA-Seq experiment. Fruit samples were collected in the 2015 season at harvest and maintained at 20°C for 3 days to accomplish shelf life. Later, samples were frozen in liquid N_2_ and stored at −80 °C until RNA extraction.

### RNA Isolation, Library Construction, and cDNA Sequencing

Total RNA was extracted using a pool of five fruit per sibling, according to Gudenschwager et al. (2012). The total RNA concentration was measured using a Qubit^®^ 2.0 Fluorometer (Invitrogen, Paisley, UK), following the manufacturer’s instruction. Sample integrity was evaluated using a Fragment Analyzer™ Automated CE System.

To accomplish the library’s construction, 10 µg of total RNA was used from each pool, and mRNA isolation was made using poly(A) beads. Subsequently, libraries were constructed using a TruSeq Stranded Total RNA Library Prep Kit (Illumina) and sequenced using a Hi-Seq 2000 Illumina platform (Macrogen, Korea). Fruit pooled samples from 12 siblings were used as biological replicates for each group (six for HRS and six for LSR). Also, two technical replicates (two independent lanes of HiSEq2000) were used per sample. The RNA-Seq data used in this study are available at the NCBI’s Sequence Read Achieve (available at: http://www.ncbi.nlm.nih.gov/sra) with SRA accession number SRP186384.

### Sequencing Data Analysis

Paired-end reads were analyzed using FastQC to evaluate read quality, and we trimmed the raw data reads by Trim Galore Cutadapt (Martin, 2011), using -paired and -q 25 options. Subsequently, trimmed reads were aligned to the reference genome for peach version 2.0 [Verde et al., 2013; Genome Database for Rosaceae (GDR)] using software STAR (Dobin et al., 2013), and later annotated. SAMtools was used to sort.bam files using the option -n (Li et al., 2009). Counts per read were calculated with HTseq-count software (Anders et al., 2015), later normalized as counts per million (CPM) (Glusman et al., 2013).

### QTL and eQTL Analysis

The QTL analysis was performed using the available linkage map of V×V (Nuñez-Lillo et al., 2015), the softening rate data determined during three consecutive seasons of evaluation (2014, 2015 and 2016) and the library ‘qtl’ of statistical software R (Broman et al., 2003). Also, chlorophyll absorbance (I_AD_ value) was used as co-variable. To identify conventional QTLs, two strategies were used: Interval Mapping test (IM) and Haley–Knott (HK) (Collard et al., 2005; Arends et al., 2010; Eduardo et al., 2013).

In the case of eQTL analysis, the mRNA transcript abundances were estimate as the Log2 of ratio between counts per million observed in Low Softening Rate siblings than counts per million observed in High Softening Rate siblings [Log2 (CPM_LSR_/CPM_HSR_)], and treated as quantitative traits that were mapped as gene-expression QTL (eQTL) (Galpaz et al., 2018).

Also, the eQTLs analysis was performed using the library ‘qtl’ of statistical software R, which provides computationally intensive algorithms based on linear regression, and suitability for experimental crosses (Broman et al., 2003). Therefore, conventional QTLs and eQTLs were analyzed by interval mapping test, and candidate QTLs or eQTLs, respectively, were selected according to LOD (logarithm of odds) score cut-off of 3 (Collard et al., 2005; Van Ooijen, 2006; Arends et al., 2010; Eduardo et al., 2013). Hence, eQTLs co-localizing with conventional QTLs were selected as candidate genes for softening rate.

### Differential Expression Analysis

To identify differentially expressed genes (DE), libraries derived from low softening rate siblings (LSR-1 to LSR-6) were compared with libraries from high softening rate HSR siblings (HSR-1 to HSR-6) at the phenological stage of harvest. Individuals exhibiting the same phenotype for softening rate were considered as biological replicates in the analysis. Differential expression analysis was done using ‘edgeR’ and statistical software R (Robinson et al., 2010). Also, results were visualized using ggplots2 and ‘heatmap.2’ function of statistical package R.

### Gene Ontology Analysis

A gene ontology (GO) enrichment analysis was performed considering all differentially expressed genes, as well as DE genes that co-localized with eQTLs. The frequency of query genes was compared with the complete reference genome for *Prunus persica* version 2.0 (http://www.rosaceae.org/), searching for possible enrichment in biological processes. Analyses were performed using the agriGO tool (available at: http://bioinfo.cau.edu.cn/agriGO), with the singular enrichment analysis and complete GO options. Significant GO terms (*p* < 0.05) were calculated using the hyper geometric distribution, and the Yekutieli multi-test adjustment method (Du et al., 2010). Subsequently, the main GO enrichment results were graphically represented using libraries ‘Clusterprofile’ (Yu et al., 2012) and ‘ggplot2’ (Wickham, 2016) of the statistical software R.

### Gene Expression Analysis by Quantitative Real-Time PCR Expression Analysis

Quantitative real-time PCR expression analysis (qPCR) of six randomly selected DE genes was performed to validate the observed *in silico* expression profiles. Hence, three DE genes derived from the group of 169 DE genes up-regulated in LSR siblings (*Prupe.1G332600*, *Prupe.3G255800* and *Prupe.8G079500*) and three derived from a group of 71 DE genes up-regulated in HSR individuals (*Prupe.1G460100*, *Prupe.5G130800* and *Prupe.7G216300*) were selected for validation of their expression profiling in LSR and HSR siblings at harvest.

RNA extraction was performed using approximately 3 g of the powder obtained from fruit cheeks, previously removing the exocarp, and homogenized in liquid nitrogen according to Gudenschwager et al. (2012). The total RNA concentration was measured using a Qubit^®^ 2.0 Fluorometer (Invitrogen, Paisley, UK), following the manufacturer’s instruction. Subsequently, cDNAs were obtained by reverse transcription reactions with 1 µg of total RNA as template, using RevertAid First Strand cDNA (Thermos) following the manufacturer instructions and oligo dT primers according to standard procedures. qPCRs were carried out using the Eco Real-Time PCR System (Illumina Inc.) equipment. The qPCR amplification reactions were performed in a total volume of 10 µl containing 1 µl cDNA (50 ng/µl), 5 µl primer mix (10 µM), 5 µl Eva Green PCR Master Mix (2×) (Applied Biosystems), and 3 µl nuclease-free water. Reactions were performed using three technical replicates, and a total of 18 observations were represented per point for LSR and HSR siblings.

The thermal cycling conditions were denaturation at 95°C for 10 min, followed by 40 cycles of template denaturation at 95°C for 15 s, primer annealing at 60°C for 15 s and extension at 72°C for 15 s, followed by a single cycle at 95°C for 15 s, at 55°C for 15 s, and finally, at 72°C for 15 s. The amplification efficiency was calculated using LingRegPCR software. The relative expression values were estimated according to the Pfaffl equation (Pfaffl, 2001). Values were normalized based on the housekeeping gene Translation elongation factor 2 (TEF2), which has been selected as a reliable reference for peach (Tong et al., 2009).

### Primer Design

Specific primers for gene validation were designed using PRIMER 3 software, according to parameters described by Taylor et al. (2010), and checked *in silico* using the Operon software (available at: http://www.operon.com/tools/oligo-analysis-tool.aspx). Primers were synthesized by Integrated DNA Technologies Inc. (Coralville, Iowa).

## Results

### Phenotypic Evaluation

The V×V mapping population (N = 138) was phenotyped for fruit quality at harvest during three consecutive seasons (from 2014 to 2016). Physiological parameters, such as titratable acidity, chlorophyll absorbance, fruit weight, soluble solids, firmness, and softening rate, were evaluated (**Supplementary Table 1**).

Results showed that the titratable acidity observed in the three seasons varied from 0.1 to 0.9, with an average of 0.5 (**Supplementary Table 1**). Also, chlorophyll absorbance (I_AD_) varied from 0.8 to 1.5, with an average of 1.13 between the three seasons. Interestingly, in the case of both parameters, no significant differences between seasons were observed (**Supplementary Table 1**).

During the three analyzed seasons, fresh fruit weight (g) ranged from 69.5 to 253.4, with averages of 134.8, 148.9, and 175.5, respectively, in seasons 2014, 2015, and 2016. In fact, significant differences in average values were found between season 2016 versus seasons 2014 and 2015 (p-value < 0.05) (**Supplementary Table 1**). In the case of soluble solid content (SSC), values varied from 8.4 to 16.4 °Brix, with averages of 13.2, 11.6, and 11.3 °Brix, respectively, in seasons 2014, 2015 and 2016. Significant differences in SSC average values were found between season 2014 versus seasons 2015 and 2016 (p-value < 0.05) (**Supplementary Table 1**). Firmness evaluation varied between 13.3 to 71.2 N, with averages of 46.7, 56.9, and 50.7 N, for the 2014, 2015, and 2016 seasons, respectively. Significant differences in average values were detected when comparing seasons 2014 and 2015 (p-value < 0.05) (**Supplementary S1**).

The analysis of softening rate was performed using 12 siblings with contrasting phenotypes: six with low softening rate (LSR) and six with high softening rate (HSR) values, considering the six siblings as biological replicates, and five fruits for each individual were evaluated in the 2014, 2015, and 2016 seasons. Results are summarized in **Supplementary Table 2**. For LSR siblings, averages varied from 17.20 to 21.16, and for HSR siblings from 78.91 to 82.98; no significant differences between individuals and seasons were identified in both cases. However, significant differences between LSR and HSR siblings were observed in the three analyzed seasons (p-value < 0.05).

**Figure 1:**
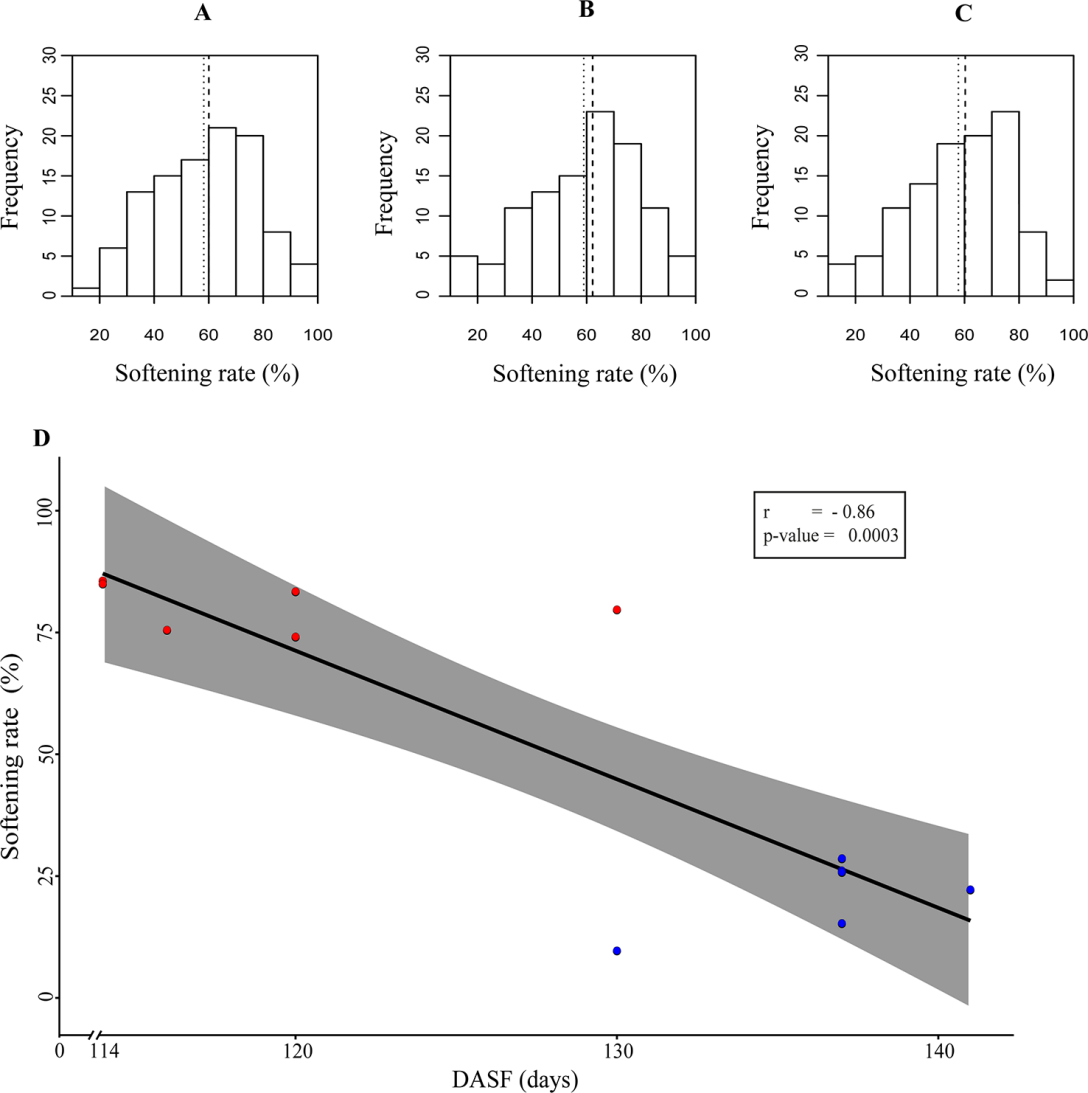
**(A**–**C)** Softening rate distribution during the analyzed seasons. The X-axis represents the softening rate percentage, while the Y-axis shows the number of siblings. The segmented line represents the median, and the dotted line represents the average. **(A**–**C)** represent the season 2014, 2015, and 2016, respectively. **(D)** Correlation analysis between softening and harvest date, the latter represented as days after September 1st (DASF), was performed using data from season 2014 based on the group of the 12 siblings with contrasting phenotypes for softening rate. High softening rate (HSR) and Low softening rate (LSR) siblings are represented in red and blue circles, respectively.

In addition, results from the characterization of the V×V population (N = 138) for softening rate showed similar average values in the three seasons, fluctuating between 41.06 and 42.24 (**Supplementary Table 2**), and followed a normal distribution (Shapiro-Wilk normality test W = 0.988, p-value = 0.514) (**Figures 1A–C**).

### QTL Analysis

**Figure 2:**
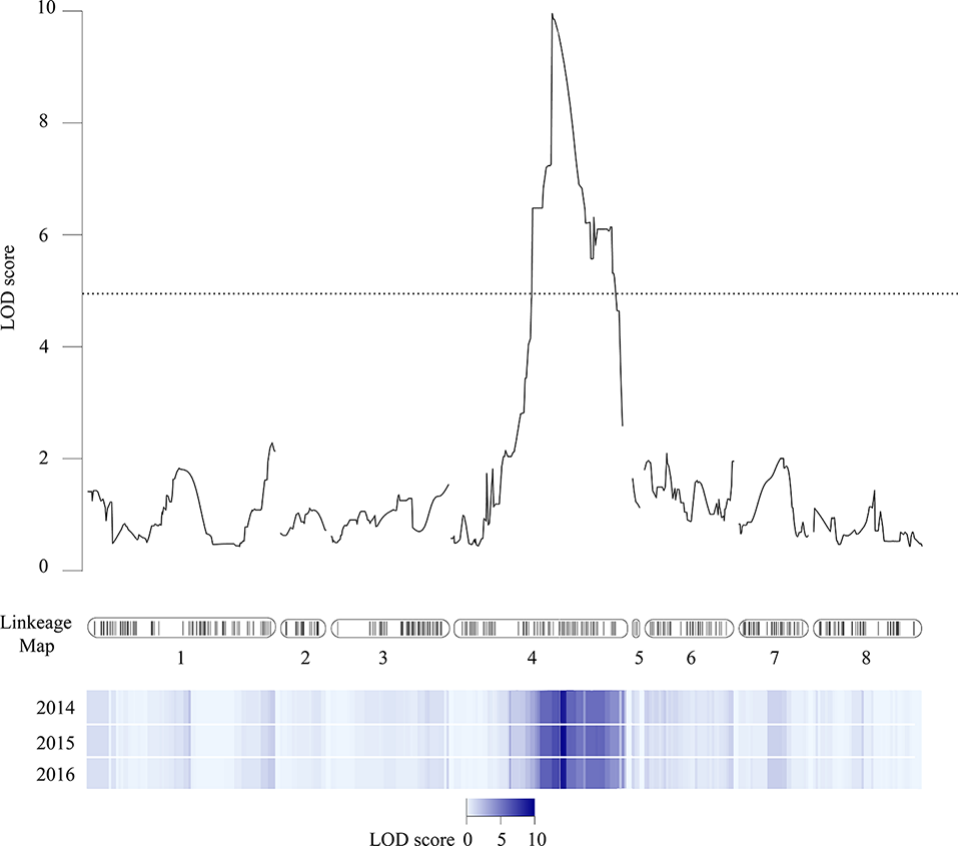
Chromosome position of qP-SOR4 at LG4. The linkage map for peach reported by Nuñez-Lillo et al. (2015) is represented in the center of the figure. Black lines correspond to markers through the eight linkage groups. LOD-score values were plotted, and results observed in analyzed seasons are shown as a heatmap (values 0 to 10).

Softening rate data from the V×V population was used to perform QTL analysis to identify chromosomic regions associated with softening rate. One QTL (qP-SOR4) with LOD = 8.9–9.6 (**Table 1**) was consistently found on LG4 with a maximum coinciding with markers SNP_IGA41265 (genome position 10,649,641 bp in chromosome 4 of the peach v2.0a1) and SNP_IGA_410398 (10.696.489 bp), located at genetic distances of 34.4 and 34.7 cM from the origin of the chromosome in V×V, respectively. A confidence interval of 27.1 to 45.2 cM was calculated using a LOD score cut-off of 5 (Nuñez-Lillo et al., 2015). qP-SOR4 showed a high effect (**Figure 2**), explaining approximately 58% of the total phenotypic variance for softening rate (**Table 1**). No additional significant QTLs were identified for this trait in any of the three evaluated seasons. The position of this QTL interval encompasses that of the *Sr* gene, also mapped in this population, and that of the NAC transcription factor gene (*PrupeG4.186800*) located at 11,116,841–11,118,655 bp of the same chromosome.

**Table 1:** QTL significantly associated with softening rate identified on LG4 during the evaluated seasons (2014, 2015 and 2016). Chromosome location, genetic position (cM), LOD score, and total phenotypical variance explained (%) are showed.

Marker	qP-SOR4 position (cM)	2014		2015		2016	
		LOD score	Phenotypic Variance explained (%)	LOD score	Phenotypic Variance explained (%)	LOD score	Phenotypic Variance explained (%)
SNP_IGA_403152	27.1	4.5	38	4.5	42	4.2	34
SNP_IGA_403613	27.4	5.8		6.0		6.1	
SNP_IGA_403741	28.2	5.8		6.0		6.1	
SNP_IGA_405773	29.3	5.8		6.0		6.1	
SNP_IGA_407364	30.4	5.8		6.0		6.1	
SNP_IGA_408059	30.7	5.8		6.0		6.1	
SNP_IGA_408505	32.6	6.3		6.8		7.0	
SNP_IGA_408981	33.3	6.3		6.8		7.0	
SNP_IGA_409379	33.6	6.3		6.8		7.0	
SNP_IGA_410265	34.4	8.9		9.6		9.1	
SNP_IGA_410398	34.7	8.9		9.6		9.1	
Slow Ripening^+^	36.5	8.6		9.3		8.8	
SNP_IGA_437516	45.2	5.8		5.9		5.3	
SNP_IGA_438108	45.6	5.9		6.0		5.4	

^+^: Slow ripening is a morphological marker on LG4 described in Nuñez-Lillo et al., 2015.

### Transcriptomic Analysis and Gene Differential Expression

An RNA-Seq experiment based on 12 siblings from the V×V population with contrasting phenotypes for softening rate was performed during 2015. Interestingly, a negative correlation between softening rate and harvest date was observed in the group of LSR and HSR siblings (Pearson correlation -0.86, p-value 0.0003) (**Figure 1D**).

**Figure 3:**
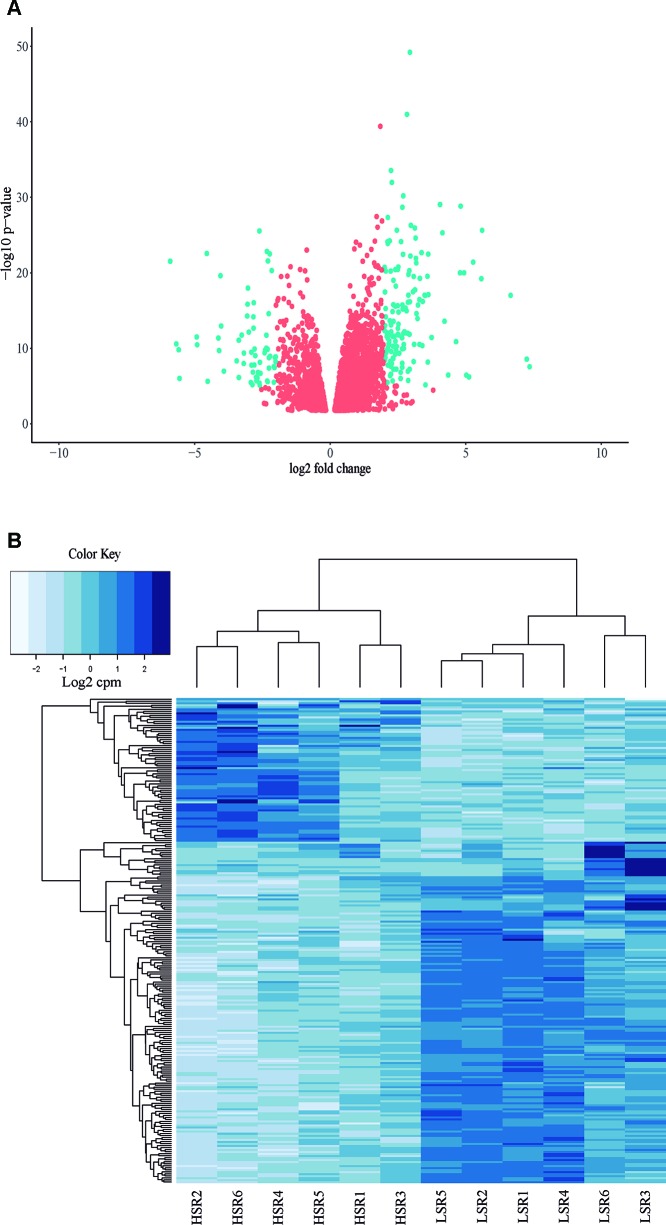
**(A)** Volcano plot of the total of 5,041 differentially expressed (DE genes) identified in the comparison between LSR and HSR siblings. **(B)** Hierarchical clustering of the selected group of 240 DE genes (logFC > 2 or logFC < -2), corresponding to 169 DE genes up-regulated in HSR siblings and 71 DE genes up-regulated in LSR siblings. Both schemes are based on gene expression normalized values.

A total of 24 libraries consisting of 12 samples with two technical replicates were analyzed (**Supplementary Figure 1**; **Supplementary Table 3**). A total of approximately 38 million paired-end reads were obtained per library, with an average length of 100 bp. Subsequently, 924 million paired-end reads were obtained, with a length of 100 bp and QC score over 37 (**Supplementary Table 3**). Thus, approximately 93.7% of total trimmed reads mapped to the reference genome of *P. persica* (**Supplementary Table 3**). Also, 14,538 genes presented at least 1 cpm (counts by million) in HSR or LSR siblings, representing 54.1% of the total genes described for peach (26,874 genes; Verde et al., 2013). A principal components analysis was performed considering the total of expressed genes in LSR and HSR libraries. The results showed that component 1 (PC1) explained 50.5% of the phenotypic variance, and two clusters were identified according to the softening rate phenotype (**Supplementary Figure 1**). In addition, a group of 5,041 differentially expressed (DE) genes between LSR and HSR individuals were identified (p-value < 0.05, FDR < 0.05), corresponding to 3,721 up-regulated and 1,320 down-regulated DE genes (**Supplementary Table 4**, **Figure 3A**).

Subsequently, a group of 240 DE genes with log_2_FC < -2, log_2_FC > 2 was selected, corresponding to 169 up-regulated DE genes and 71 down-regulated DE genes. Hierarchical clustering was performed using gene expression normalized values, and two main clusters were observed (**Figure 3B**).

**Figure 4:**
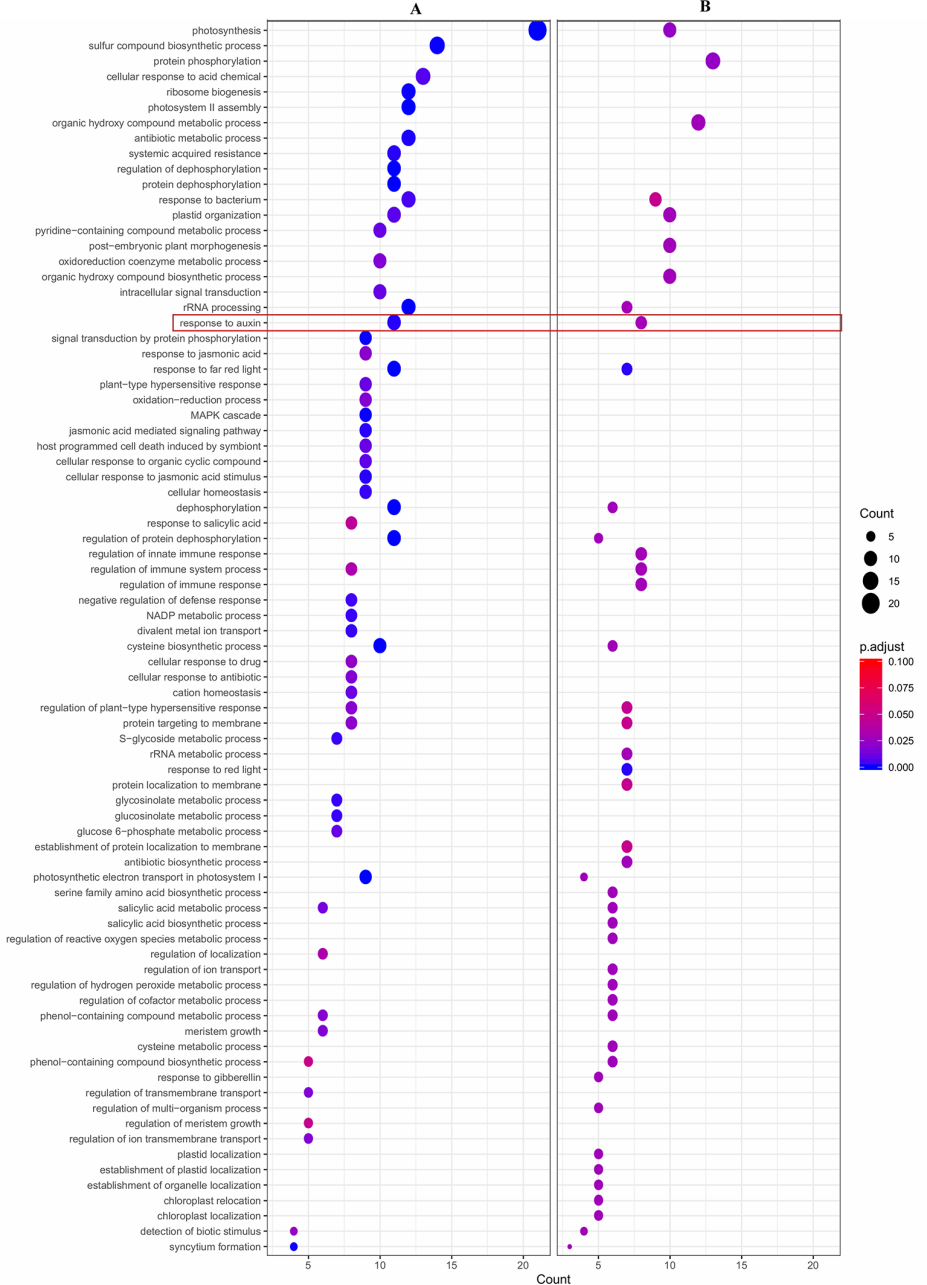
Dot plot of enriched Gene Ontology (GO) terms of **(A)** 169 differential expressed (DE) genes (logFC > 2), up-regulated in LSR siblings; and **(B)** 133 differentially expressed (DE) genes with an eQTL peak over 5 and also co-localize with qP-SOR4, identified at chromosome 4. Y-axis indicates the GO term and X-axis shows the count of genes per GO term. Color gradient indicates adjusted p-value (0.05), using the Benjamini-Hochberg method. GO term ‘Response to auxin’ (GO:0009733) is marked in a red square.

Also, a functional enrichment analysis (Gene Ontology) was developed to assess the main processes over-represented in each group of DE genes using the agriGO platform. Concomitantly, GO analysis of the group of 169 DE genes up-regulated in LSR siblings showed two over-represented categories ‘Response to auxin’ and ‘Photosynthesis’ related to the Biological Process category (**Figure 4A** and **Supplementary Figure 2**). Results are concordant with the identification of nine DE genes, which presented GO term ‘GO:0009733, Response to auxin’, all of them up-regulated in LSR siblings; four of them coding for auxin-induced proteins X10A (*Prupe.8G081800*, *Prupe.8G078700* and *Prupe.8G079600*) and 10A5 (*Prupe.8G081400*), as well as five coding for indole-3-acetic acid-induced protein ARG7 (*Prupe.8G081100*, *Prupe.8G080800*, *Prupe.8G079500*, *Prupe.8G081300*, and *Prupe.8G079200*). However, in the case of the group of 71 DE genes up-regulated in HSR individuals, no over-represented category was identified.

Furthermore, DE genes coding for YUCCA flavin mono-oxygenase (PpYUC11) (*Prupe.6G157400*, *Prupe.6G157500*) and NAC transcription factor (*Prupe.4G186800*), all of them associated to the auxin biosynthesis pathway (Serra et al., 2017), were over-expressed in HSR siblings (**Supplementary Table 4**).

Also, cell wall remodeling enzyme genes coding for endopolygalacturonases, PpendoPGM (*Prupe.4G262200*), and PpendoPGF (*Prupe.4G261900*) were found differentially expressed between LSR and HSR individuals, being over-expressed in HSR (**Supplementary Table 4**). Also, genes associated with cell wall remodeling coding for cellulose synthase-like B4 (*Prupe.3G214400*), expansin A1 (*Prupe.1G276700*) and xyloglucan endotransglucosylase/hydrolase 30 (*Prupe.5G027800*) were also identified in the group of genes co-localizing with qSOR4 (**Supplementary Table 5**).

### eQTLs Analysis

An eQTL analysis was performed, and 2,413 eQTLs exhibited LOD scores between 3.0 and 19.9, associated with 1,496 genes, and distributed through the eight peach chromosomes. Of these eQTLs, 409 showed LODs >5.0. Subsequently, to identify candidate genes associated with softening rate in peach, a possible co-localization of QTLs and eQTLs was analyzed by the comparison of LSR and HSR siblings. Hence, a group of 1,062 eQTLs exhibiting a LOD >5.0 were found co-localizing at LG4 (27.1–45.2 cM). In relation to the linear regression analyses implemented into R/qtl software to identify differentially expressed genes overlapping at qP-SOR4, several algorithms were evaluated obtaining the same results.

**Figure 5:**
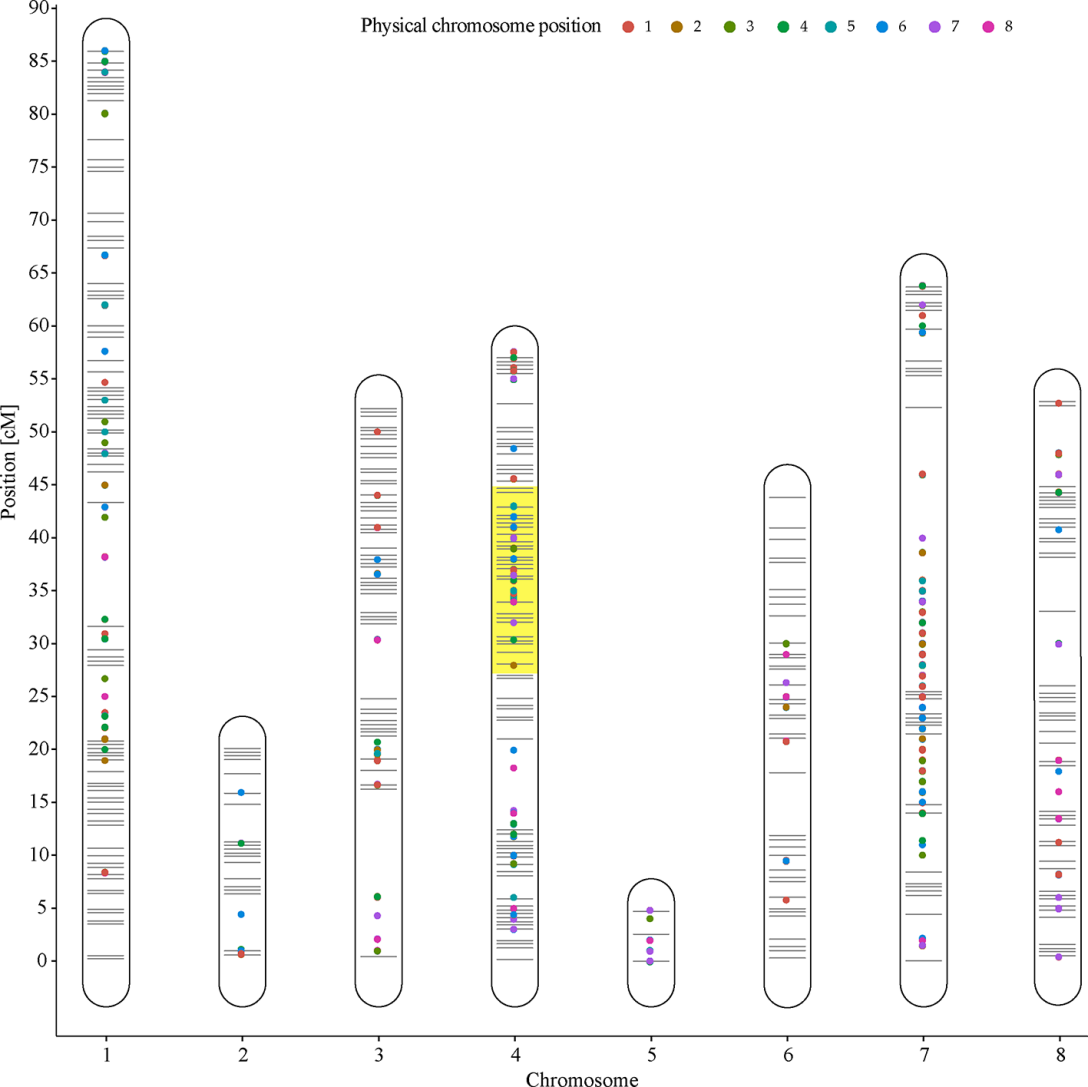
Expression QTLs (eQTL) distribution on Venus × Venus genetic linkage map. Lines represent eight chromosomes of peach. The co-localization of eQTLs (LOD score >5) on qP-SOR4 is highlighted in yellow.

Furthermore, 133 DE genes were found co-localizing with qP-SOR4 identified on LG4 (**Supplementary Table 5**) exhibiting a LOD score greater than 5 (**Figure 5**), of which 13 correspond to cis QTLs (10%) and 120 to trans QTLs (90%). Also, a functional enrichment analysis (Gene Ontology) was performed considering this subset of genes. Thus, ‘Response to auxin’ was the main over-represented category related to the Biological Process category (**Figure 4B**; **Supplementary Figure 3**), in agreement with the identification of DE genes coding for proteins related to perception or response to auxin, such as indole-3-acetic acid (IAA)-inducible genes (Aux/IAA, SAUR) (**Figure 4B**; **Supplementary Figure 3**). Additionally, we identified five genes coding for SAUR-like auxin-responsive protein family (*Prupe.8G081400*, *Prupe.8G079200*, *Prupe.8G081300*, *Prupe.8G078700*, and *Prupe.8G081100*), and an early auxin response gene, capable of being induced by auxin and ethylene (Li et al., 2015) (**Supplementary Figure 4**).

### qPCR Expression Analysis of a Group of Differentially Expressed Genes From a Comparison of Siblings With Contrasting Phenotypes for Softening Rate

**Figure 6:**
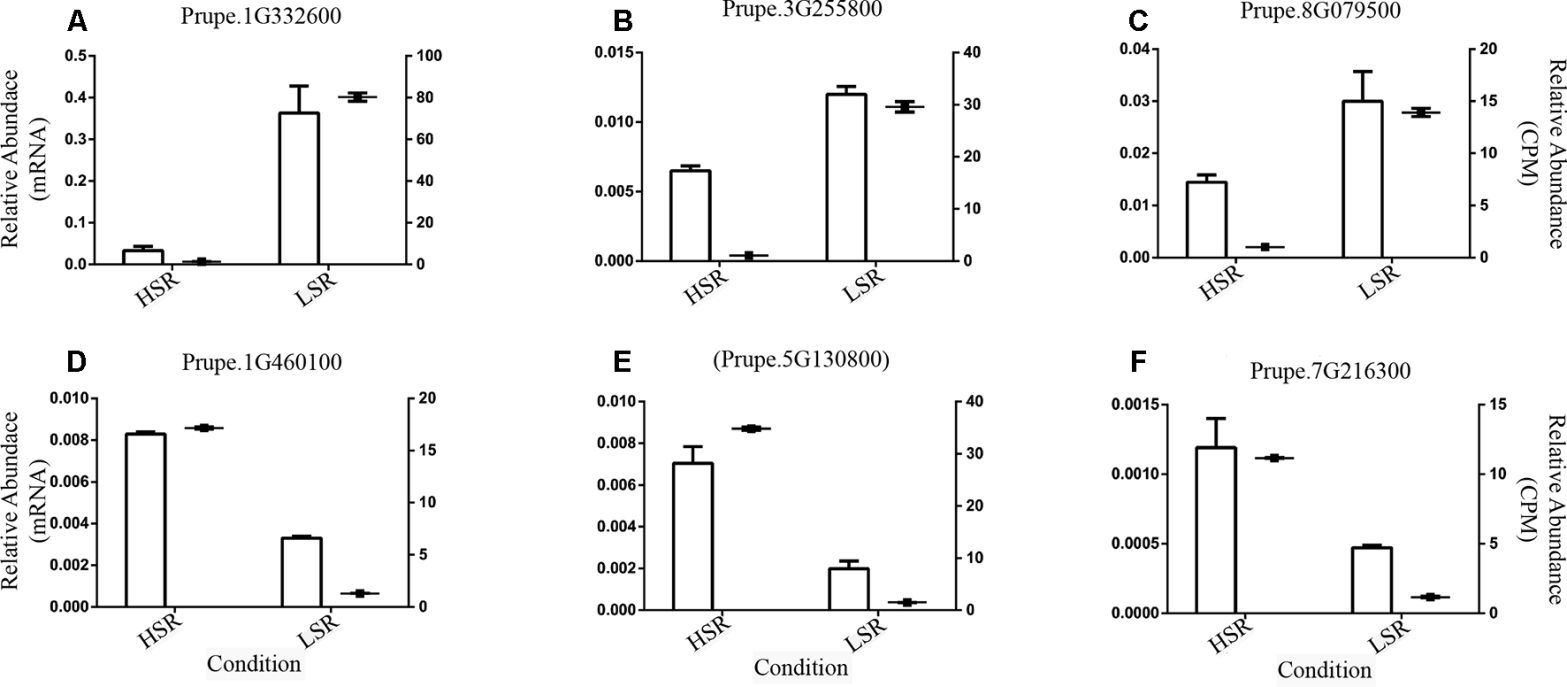
Validation of six differentially expressed (DE) genes randomly selected; identified in the comparison between LSR and HSR siblings; using real-time (qPCR) experiments at harvest. Genes are **(A)** RNA-dependent RNA polymerase (*Prupe.1G332600*); **(B)** Cyclin-like family protein (*Prupe.3G255800*); **(C)** Indole-3-acetic acid-induced protein ARG7 (*Prupe.8G079500*); **(D)** Unknown Protein Function (*Prupe.1G460100*); **(E)** Unknown Protein Function (*Prupe.5G130800*); and **(F)** Inositol oxygenase 4 (*Prupe.7G216300*). The TEF2 gene (possible Translation elongation factor 2 gene) was used as a reference gene, and gene expression was expressed as relative expression. The relative abundance based on qPCR (Y-left axis) was compared with the relative abundance of normalized gene expression determined by RNA-Seq experiment (Y-right axis); both values are respectively represented as bar and point, and the standard deviation was also plotted.

Specific primers were designed to validate six randomly selected DE genes. The primers used in real-time experiments (qRT-PCR) are summarized in **Supplementary Table 6**. Subsequently, the identified transcriptional expression profiles were validated by real-time qPCR experiments at harvest (**Figure 6**).

The selected DE genes encompassed RNA-dependent RNA polymerase (*Prupe.1G332600*), Cyclin-like family protein (*Prupe.3G255800*), Indole-3-acetic acid-induced protein ARG7 (*Prupe.8G079500*), two genes coding for an Unknown Protein Function (*Prupe.1G460100*, *Prupe.5G130800*), and Inositol oxygenase 4 (*Prupe.7G216300*). The obtained results agreed with the *in silico* expression profiles observed in LSR and HSR siblings at harvest (**Figure 6**).

In fact, the expression profile of the DE gene coding for indole-3-acetic acid-induced protein ARG7 (*Prupe.8G079500*), a possible auxin homeostasis-related gene (**Figure 6**), was confirmed by qPCR at harvest. This evidence suggests that auxin homeostasis is altered in LSR individuals and might be associated with low auxin levels, which might activate a compensatory mechanism to accomplish softening.

## Discussion

### Peach Fruit Softening

Peaches and nectarines are frequently maintained for 3–4 weeks after harvest, which could significantly affect softening. For this reason, the responsiveness to postharvest conditions represents a key quality trait for breeding programs (Ramina et al., 2008). However, the genetic basis of postharvest life in peaches is hampered by the difficulties associated with its phenotyping (Bassi and Monet, 2008).

Previous studies developed by Nuñez-Lillo et al. (2015) and Serra et al. (2017), which were performed to understand fruit quality traits related to softening, mainly focused on the maturity date or the identification of conventional QTLs associated with the slow melting flesh character (SMF). To provide additional information on this character, we studied the genetic factors associated with fruit softening rate (SOR), integrating data derived from conventional QTLs and eQTLs in an F2 mapping population of *P. persica*. We observed a normal distribution for SOR in this progeny in the three years studied, suggesting that it is under polygenic control (**Figures 1A–C**).

Firmness loss values were analyzed in a subset of siblings with contrasting phenotypes for softening rate, denominated as LSR and HSR, and results for SOR were consistent for this sample among seasons (**Supplementary Table 2**). These results were similar to those previously reported, where SOR values were used to estimate the SMF trait, considered as a slower fruit softening after the maturity stage (Serra et al., 2017). In fact, the SOR values for the SMF trait in LSR siblings (17.2–21.2) were similar to those previously reported by Serra et al. (2017) in the SMF variety ‘Big Top’ and other siblings considered SMF (< 20.0). In fact, SMF fruit characterization also presents some difficulties and evidence suggests that it is under oligogenic or polygenic control, with at least two QTLs involved, one on LG4 and another on LG5 (Serra et al., 2017).

Firmness loss values observed in MF varieties ‘Armking’ (62.5–75.3) and ‘Nectaross’ (64.2–65.0) followed a similar pattern as HSR siblings (78.9–82.98) (Serra et al., 2017). Furthermore, segregating populations derived from the cross of ‘Big Top’ and ‘Armking’ (Bt×Ak) or ‘Nectaross’ (Bt×Nr) exhibited average values of firmness loss of 43.62 and 40.25, similar to those obtained in the V×V mapping population in analyzed seasons (41.06–42.24). Overall, these data suggest that the populations previously analyzed had a similar fruit softening behavior than V×V and that firmness loss is a robust approach to measure fruit SOR as a component of postharvest shelf life of peach fruits.

### QTL Analysis

A single major QTL (qP-SOR4), explaining 34–42% of the phenotypic variance for the trait, was found in the progeny studied. This QTL maps at a different position than the *M* locus, and co-locates with SOR-related traits such as the (*Sr/sr*) gene, responsible for slow ripening (SMF), that segregates in the V×V progeny (Eduardo et al., 2015; Nuñez-Lillo et al., 2015), and the (*MD*/*md*) gene that determines maturity date (Cantín et al., 2010; Eduardo et al., 2011; Dirlewanger et al., 2012; Pirona et al., 2013; Donoso et al., 2016; Serra et al., 2017). Hence, a NAC transcription factor located within LG4 interval (encoded by *Prupe.4G186800*) has been proposed as the causal gene for *MD* and *Sr* (Pirona et al., 2013; Eduardo et al., 2015). Also, qP-SOR4 maps with a QTL previously reported for firmness loss and maturity date in peach cultivars ‘Armking’ and ‘Nectaross’, both MF types (Serra et al., 2017).

Interestingly, this region of LG4 seems to be a chromosomal hotspot where major genes or major QTLs explaining important traits have been identified in peach and other *Prunus* crops. In addition to traits related to SOR, such as maturity date and SMF, others related to fruit quality have been described, such as fruit weight (Da Silva Linge et al., 2015; Hernandez Mora et al., 2017), sugar contents (Zeballos et al., 2016; Hernandez Mora et al., 2017), mesocarp thickness (Donoso et al., 2016), and mealiness (Cantín et al., 2010; Nuñez-Lillo et al., 2015), as well as disease resistance genes, including brown rot (Martínez-García et al., 2013; Pacheco et al., 2014) and bacterial spot (Yang et al., 2013).

Also, QTLs related to phenology and fruit quality traits have been reported at LG4 in apricot, such as firmness and soluble solids (Salazar et al., 2014), as well as ripening time and skin color in Japanese plum (Salazar et al., 2017; García-Gómez et al., 2019). While some of these traits might result from pleiotropic effects associated with the same gene or genes (Eduardo et al., 2011; Eduardo et al., 2015; Serra et al., 2017), the importance of this genomic region is evident and deserves additional study.

Furthermore, one QTL for firmness in apple was identified using a phenotyping method able to discriminate between the acoustic and mechanical components of the texture. The QTL is located on chromosome 10 (Longhi et al., 2012). Considering the macrosynteny in members of the *Rosaceae* family, the study of *Malus domestica*, *Fragaria vesca*, and *P. persica* showed a high level of synteny among chromosomes 4 of *Prunus* and 10 of *Malus* (Illa et al., 2011; Jung et al., 2012). This evidence agreed with our finding of a QTL associated with softening rate on chromosome 4, and might support the hypothesis that fruit texture is determined by common genes in different members of the *Rosaceae* family (Illa et al., 2011).

The results obtained in this work could lead to the development of molecular markers associated with quality traits, such as SOR, which could be integrated into marker-assisted selection (MAS), based on their transferability along *Prunus* family, and also contribute to breeding efforts (Salazar et al., 2017; García-Gómez et al., 2019).

### Candidate Genes Associated With SMF Trait

It has been proposed that IAA concentration might control ethylene production (Tatsuki et al., 2013) and the fruit softening process in peaches (Pan et al., 2015). It seems that auxin is involved in a complex interplay with ethylene during peach ripening (Trainotti et al., 2007; Liu et al., 2015). The expression of ethylene biosynthesis and signaling genes is regulated in fleshy fruits (e.g., tomato and peach) by auxin, where their texture is greatly modified during ripening (Gillaspy et al., 1993; Jones et al., 2002; Trainotti et al., 2007; Pirrello et al., 2012; Liu et al., 2015). Hence, the concentration of auxin increases before ripening in peach fruit coinciding with the climacteric ethylene peak (Tatsuki et al., 2013).

Concomitantly, auxin regulates the expression of Auxin Response Factors (ARF) and Aux/IAA encoding genes, associated to the auxin signaling pathway, whose expression increases in the peach mesocarp during ripening (Jones et al., 2002; Trainotti et al., 2007). Additionally, tomato fruit firmness is partially modulated by a transcription factor related to auxin-responses (SlARF4), reinforcing its possible role in ripening control in climacteric fruits (Liu et al., 2015).

Moreover, auxin induces the expression of genes encoding 1-aminocyclopropane-1-carboxylic acid (ACC) synthase, associated with the ethylene biosynthesis (Tatsuki et al., 2013). Also, exogenous application of ethylene on peach fruit has been shown to promote postharvest softening (Haji et al., 2001; Hayama et al., 2006).

Our results show that DE genes coding for YUCCA flavin mono-oxygenase (*PpYUC11*) (*Prupe.6G157400*, *Prupe.6G157500*) and NAC transcription factor (*Prupe.4G186800*) – all of them associated to the auxin biosynthesis way (Mashiguchi et al., 2011; Serra et al., 2017) – were over-expressed in HSR siblings (**Supplementary Table 4**). In fact, the *Prupe.4G186800* was proposed as a candidate for a maturity date gene (Pirona et al., 2013) and for the slow ripening gene (Eduardo et al., 2015) that co-locates with the position of qP-SOR4 identified in this work. Additionally, the expression profile of the DE gene coding for indole-3-acetic acid-induced protein ARG7 (*Prupe.8G079500*) was experimentally confirmed by qPCR, and was also up-regulated in LSR siblings. Our findings were concordant with previous reports of high levels of auxin-induced cell wall modifications promoting fast softening rate in peach fruits (Tatsuki et al., 2013).

Also, stony hard (SH) peach cultivars, such as ‘Yumyeong’, ‘Xia cui’, and ‘CN16’, showed a deficient auxin biosynthesis, probably resulting from low levels of *PpYUC11* gene expression, which might reduce the level of IAA in the stony hard phenotype (Pan et al., 2015). The flavin mono-oxygenase gene *PpYUC11* has also been proposed as a regulator of the concentration of IAA at the late-ripening stage in peach (Pan et al., 2015). In fact, a higher expression level and stronger up-regulation of *PpYUC11* during maturation to the ripening stage had been correlated with IAA concentration as well as with the activation of the ethylene biosynthesis mediated by the *PpACS1* gene; concomitantly, a rapid fruit softening at the late-ripening stage in MF peach cultivars is observed (Tatsuki et al., 2013; Pan et al., 2015).

This result could be supported by previous reports from comparisons made during the fruit ripening stage of melting and stony hard (SH) peach varieties, in which the latter was characterized by the absence of ethylene production and fruit softening. In fact, differential expression of several auxin-homeostasis-related genes, as well as a differential IAA accumulation, were observed, associated to low IAA concentration in the SH peach variety (Haji et al., 2003; Haji, 2014; Pan et al., 2015).

Additionally, the process of cell wall disassembly has been considered responsible for softening and textural changes on fruit during ripening (Brummell and Harpster, 2001). The widely reported involvement of polygalacturonase (PG), an important hydrolytic enzyme involved in pectin degradation during fruit softening (Qian et al., 2016), likely plays a key role as a positive regulator mediated by ethylene (Hiwasa et al., 2003; Asif and Nath, 2005).

Moreover, we found that cell wall remodeling enzyme genes coding for endopolygalacturonases, PpendoPGM and PpendoPGF – previously reported as associated to the MF/NMF and clingstone/freestone, respectively (Gu et al., 2016) – were over-expressed in HSR individuals (**Supplementary Table 4**). Furthermore, based on the comparison of peach cultivars presenting fast (‘Qian Jian Bai’) and slow softening patterns (‘Qin Wang’), it has been proposed that candidate genes coding for PGs might play major roles in peach fruit softening (Qian et al., 2016).

Also, genes associated with cell wall remodeling coding for cellulose synthase-like B4 (*Prupe.3G214400*), expansin A1 (*Prupe.1G276700*), and xyloglucan endotransglucosylase/hydrolase 30 (*Prupe.5G027800*) were also identified in the group of genes co-localizing with qP-SOR4 at LG4 (**Supplementary Table 5**). In fact, expansin and XTH are active drivers of cell wall remodeling, both of which are sensitive to auxin induction (Labavitch and Ray, 1974; Nishitani and Masuda, 1981).

By an integrated analysis based on QTLs, eQTLs, and transcriptomic profiling, we identified genes involved in the metabolic pathways affecting fruit softening rate in peach. Some of these genes were selected as candidates possibly acting as key drivers of this trait in peach cultivars. These results are of interest to efforts to better understand the process of fruit maturation in peach and to estimate the effects of the natural allelic variation for these genes.

This information can be used to design appropriate breeding strategies to improve the postharvest performance of commercial peach cultivars, considering the high synteny described in *Prunus*, which could facilitate a successfully transferability of molecular markers suitable to be integrated in breeding selection programs (Dirlewanger et al., 2004; Infante et al., 2008).

## Conclusions

We have developed an integrative analysis involving conventional QTLs, eQTLs, and transcriptome profiling analysis focused on siblings with contrasting phenotypes for flesh softening in peach fruit. A conventional QTL was identified at LG4 co-localizing with a previously reported QTL associated with fruit firmness and the slow melting flesh character (Serra et al., 2017).

The integration of differential gene expression, as well as conventional and expression QTL analysis, provided a wide characterization of possible genetic factors affecting the regulation of softening rate character in peach. Our findings suggest that genes associated with the auxin biosynthetic pathway were up-regulated in HSR siblings, reinforcing the importance of auxin as a key driver of rapid fruit softening at the late-ripening stage in melting flesh peach cultivars. Conversely, the LSR phenotype might be explained by an altered auxin-homeostasis associated with low auxin levels. This work contributes to unraveling the genetic mechanisms responsible for the softening rate in peaches and nectarines. Furthermore, findings derived from this study could lead to the development of molecular markers associated with softening rate. Considering the high synteny described in *Prunus*, this might facilitate their successful transference and integration into MAS, which would be useful for breeding purposes.

## Data Availability Statement

Publicly available datasets were analyzed in this study. This data can be found here: www.ncbi.nlm.nih.gov/sra.

## Author Contributions

TC-V performed QTL analysis, eQTL analysis, and RNA-Seq analysis. CM-E contributed with RNA-Seq, GO, and KEGG analysis and writing the manuscript. AR carried out the gene validation by RT-qPCR and KEGG analysis. RC-V, RP, and PA collaborated to writing-review and editing of the manuscript. CM supervised the bioinformatic analysis and worked on writing-original draft.

## Funding

This work was supported by Fondo Nacional de Desarrollo Científico y Tecnológico (FONDECYT) 1160584, FONDEF D13i10005 and CORFO Biofrutales 13CTI21520-SP04.

## Conflict of Interest

The authors declare that the research was conducted in the absence of any personal, professional or financial relationship that could constitute a conflict of interest.
