# Supplementary material for: Expression QTL (eQTLs) Analyses Reveal Candidate Genes Associated With Fruit Flesh Softening Rate in Peach [Prunus persica (L.) Batsch]
Source: Front Plant Sci. 2019 Dec 3;10:1581. doi: 10.3389/fpls.2019.01581 (PMC6901599; doi:10.3389/fpls.2019.01581)
Supplement: Supplementary file 1 [file DataSheet_1.pdf]

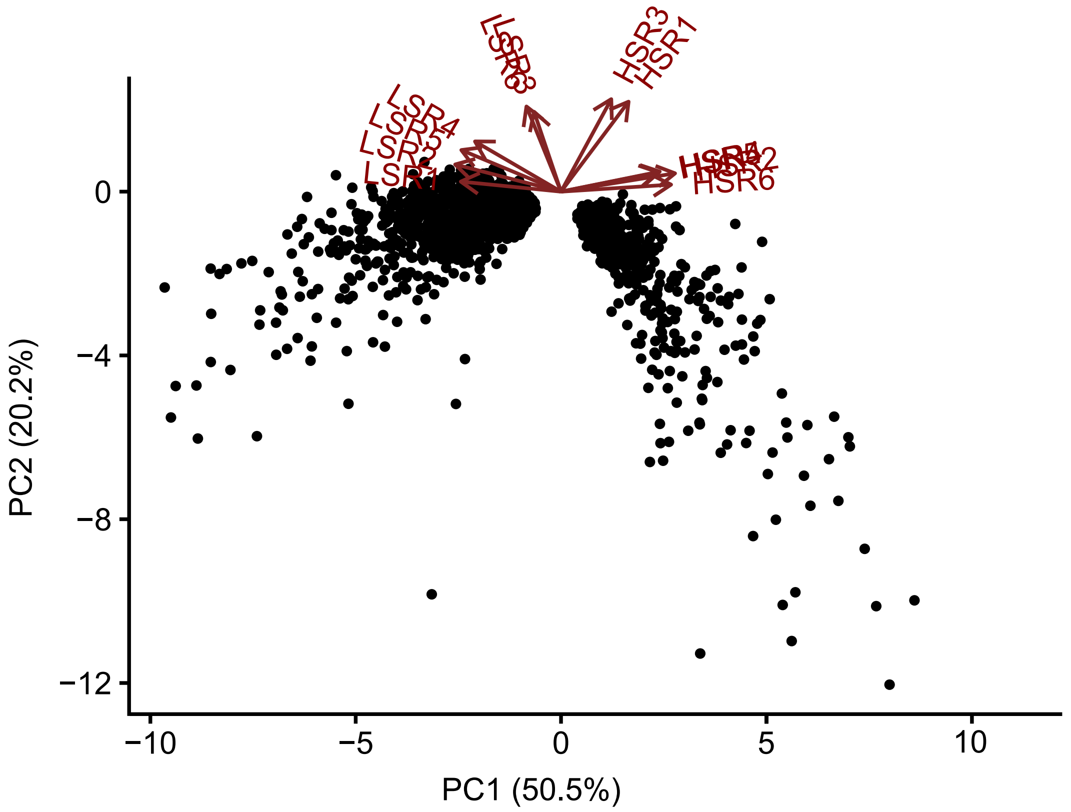

Supplementary Figure 1. Principal component analysis (PCA) using the total of expressed genes in LSR and HSR libraries and normalized expression data (CPM). The result shows that component 1 (PC1) explains 50.5 % of the phenotypic variance.
