# Supplementary material for: Expression QTL (eQTLs) Analyses Reveal Candidate Genes Associated With Fruit Flesh Softening Rate in Peach [Prunus persica (L.) Batsch]
Source: Front Plant Sci. 2019 Dec 3;10:1581. doi: 10.3389/fpls.2019.01581 (PMC6901599; doi:10.3389/fpls.2019.01581)
Supplement: Supplementary file 2 [file DataSheet_2.pdf]

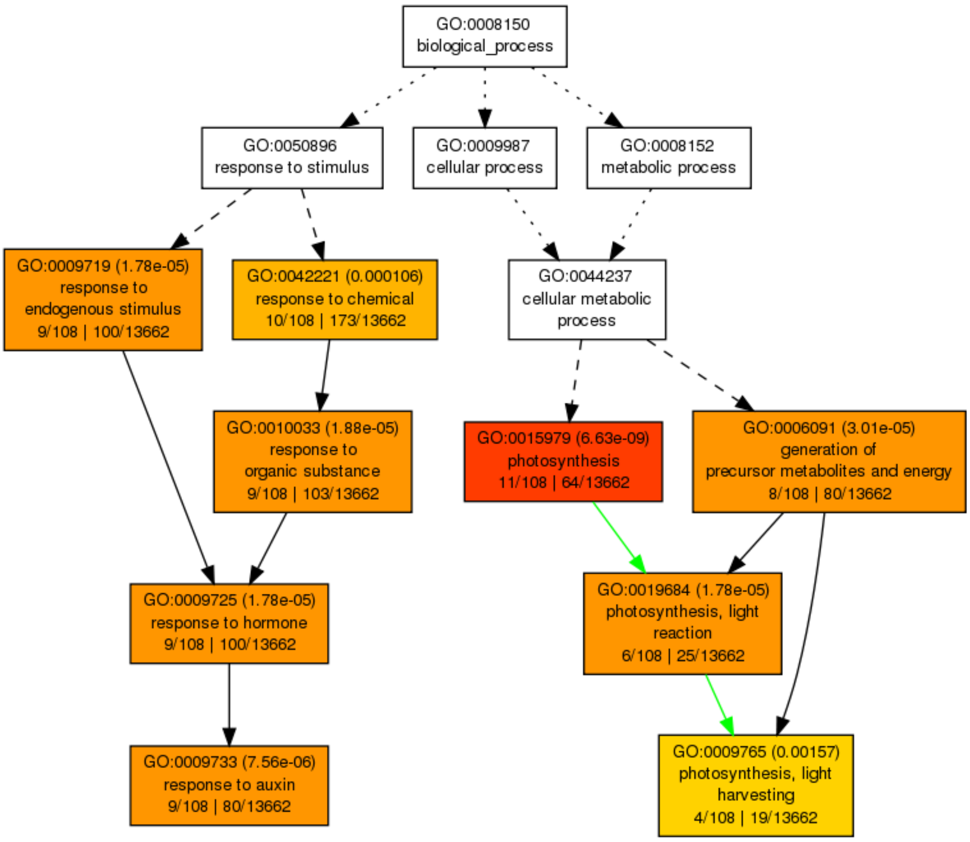

Supplementary Figure 2. GO enrichment of 169 differential expressed (DE) genes with logFC higher than 2; over-expressed in LSR siblings. Biological Process category is shown; and only significantly over-represented categories were considered ($p < 0.05$ and FDR < 0.05). The analysis was performed using the online agriGO tool (available at http://bioinfo.cau.edu.cn/agriGO) and the GO complete category. The boxes contain the GO number; the p-value (in parentheses); the category description; the number of genes in each category associated with the GO term versus the total of query genes and the number of genes in each category out of 13,662 genes of the reference genome of *Prunus persica* version 2.0 (available at http://www.rosaceae.org/); with associated GO terms. The arrows indicate the relationships among the GO categories; as follows: black solid arrows mean that a GO category is also included in the other one; red solid arrows mean that one GO category positively regulates the other; green solid arrows mean that the GO category negatively regulates the other; black dashed arrows indicate that there are two significant nodes related to the GO category; and black dotted arrows indicate that only one significant node is related to the GO category.
