# Supplementary material for: Expression QTL (eQTLs) Analyses Reveal Candidate Genes Associated With Fruit Flesh Softening Rate in Peach [Prunus persica (L.) Batsch]
Source: Front Plant Sci. 2019 Dec 3;10:1581. doi: 10.3389/fpls.2019.01581 (PMC6901599; doi:10.3389/fpls.2019.01581)
Supplement: Supplementary file 4 [file DataSheet_4.pdf]

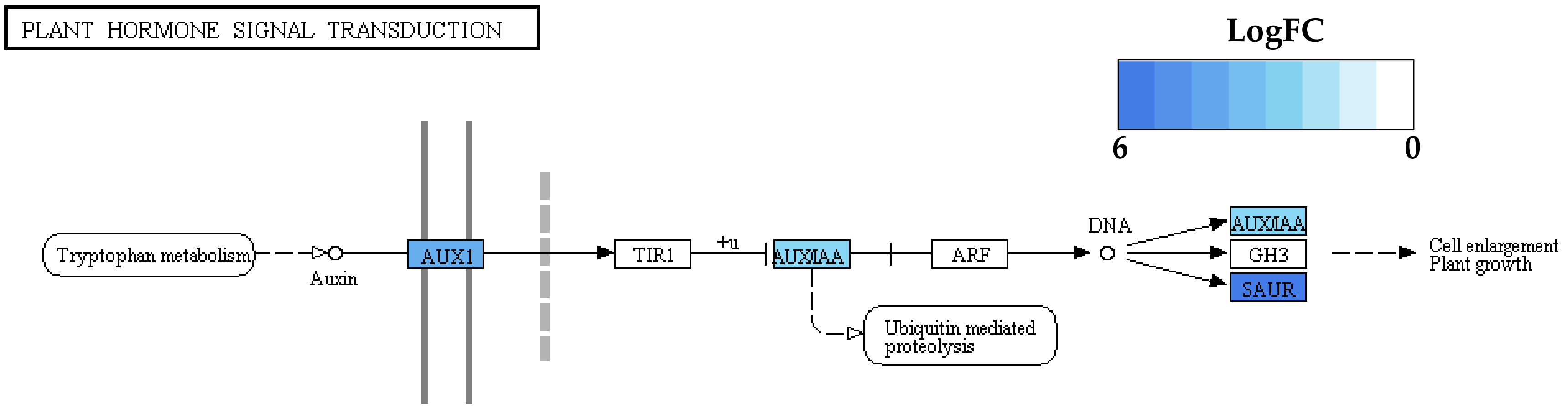

Supplementary Figure 4. Pathway KEGG analysis of plant hormone signal transduction related to tryptophan metabolism and auxin biosynthesis. Candidate gene expression is represented as logFC derived from a comparison of LSR versus HSR siblings.
