# Supplementary material for: Expression QTL (eQTLs) Analyses Reveal Candidate Genes Associated With Fruit Flesh Softening Rate in Peach [Prunus persica (L.) Batsch]
Source: Front Plant Sci. 2019 Dec 3;10:1581. doi: 10.3389/fpls.2019.01581 (PMC6901599; doi:10.3389/fpls.2019.01581)
Supplement: Supplementary file 5 [file Table_1.docx]

**Supplementary Table 1.** Phenotypical characterization of physiological parameters at harvest of Venus x Venus F2 population. Measurements were recorded during seasons 2014, 2015 and 2016. N represents the total of siblings used in the phenotypic characterization varied from 104 to 113 plants, depending of the evaluated season.

| **Trait** | **Season** | **N** | **Means** | | **Max** | **Min** | **SD** |
| --- | --- | --- | --- | --- | --- | --- | --- |
| Firmness (N) | 2014 | 113 | 46.7 | a | 66.7 | 13.3 | 13.8 |
|  | 2015 | 104 | 56.9 | b | 71.2 | 29.8 | 8.9 |
|  | 2016 | 107 | 50.7 | ab | 68.9 | 22.2 | 17.8 |
| I_AD_ | 2014 | 113 | 1.1 | a | 1.5 | 0.8 | 0.1 |
|  | 2015 | 104 | 1.2 | a | 1.5 | 0.8 | 0.1 |
|  | 2016 | 107 | 1.1 | a | 1.3 | 0.8 | 0.1 |
| Weight (g) | 2014 | 113 | 134.8 | a | 253.4 | 69.5 | 32.2 |
|  | 2015 | 104 | 148.9 | a | 234.6 | 95.0 | 29.9 |
|  | 2016 | 107 | 175.5 | b | 252.7 | 90.7 | 32.6 |
| SSC^†^ (°Brix) | 2014 | 113 | 13.2 | b | 16.4 | 9.7 | 1.4 |
|  | 2015 | 104 | 11.6 | a | 15.0 | 8.4 | 1.4 |
|  | 2016 | 107 | 11.3 | a | 15.7 | 9.1 | 1.3 |
| TA^§^ (%) | 2014 | 113 | 0.5 | a | 0.9 | 0.1 | 0.2 |
|  | 2015 | 104 | 0.5 | a | 0.8 | 0.1 | 0.2 |
|  | 2016 | 107 | 0.5 | a | 0.9 | 0.1 | 0.2 |

Different letters indicate means ± standard deviation of the mean (SD) with statistically significant differences for each trait between among seasons according to Tukey test (p < 0.05). I_AD_= chlorophyll absorbance; ^†^Soluble solids content; ^§^Titratable acidity (%).
