# Supplementary material for: Expression QTL (eQTLs) Analyses Reveal Candidate Genes Associated With Fruit Flesh Softening Rate in Peach [Prunus persica (L.) Batsch]
Source: Front Plant Sci. 2019 Dec 3;10:1581. doi: 10.3389/fpls.2019.01581 (PMC6901599; doi:10.3389/fpls.2019.01581)
Supplement: Supplementary file 6 [file Table_2.docx]

**Supplementary Table 2.** Softening rate at harvest of the selected sibling from V×V population exhibiting contrasting phenotypes. Each value corresponded to mean phenotypic values during seasons 2014, 2015 and 2016.

| **VxV**  **Sibling** | **Phenotypic evaluated seasons** | | | | | |
| --- | --- | --- | --- | --- | --- | --- |
|  | **2014** |  | **2015** |  | **2016** |  |
| ^+^LSR1 | 26.01 |  | 17.41 |  | 22.65 |  |
| LSR2 | 28.48 |  | 25.98 |  | 24.27 |  |
| LSR3 | 9.58 |  | 10.43 |  | 10.75 |  |
| LSR4 | 22.05 |  | 12.07 |  | 15.15 |  |
| LSR5 | 25.69 |  | 23.15 |  | 32.28 |  |
| LSR6 | 15.19 |  | 14.15 |  | 23.89 |  |
| LSR mean | 21.16 |  | 17.20 |  | 19.75 |  |
| *HSR1 | 75.39 |  | 80.48 |  | 78.92 |  |
| HSR2 | 73.97 |  | 81.96 |  | 78.48 |  |
| HSR3 | 79.58 |  | 80.28 |  | 76.17 |  |
| HSR4 | 84.87 |  | 85.56 |  | 79.17 |  |
| HSR5 | 83.30 |  | 84.49 |  | 80.34 |  |
| HSR6 | 85.53 |  | 85.08 |  | 80.37 |  |
| HSR mean | 80.44 |  | 82.98 |  | 78.91 |  |
| † Population mean | 41.81 |  | 41.06 |  | 42.24 |  |

^+^ LSR, ‘Low Softening Rate’; *HSR, ‘High Softening Rate’.

^†^ Population mean corresponds to the observed softening rate mean in the ‘VxV’ population for each season.
