# Supplementary material for: Expression QTL (eQTLs) Analyses Reveal Candidate Genes Associated With Fruit Flesh Softening Rate in Peach [Prunus persica (L.) Batsch]
Source: Front Plant Sci. 2019 Dec 3;10:1581. doi: 10.3389/fpls.2019.01581 (PMC6901599; doi:10.3389/fpls.2019.01581)
Supplement: Supplementary file 7 [file Table_3.docx]

**Supplementary Table 3.** Read mapping summary considering a total of 24 libraries. Total of reads, the percentage of reads mapped to reference genome of *P. persica* (GDR, available at: <http://www.rosaceae.org/>), and the total expressed genes per library (gene expression > 2 CPM) are described.

| **Sibling** | **Total of reads** | **Mapped reads [%]** | **Total of expressed genes** |
| --- | --- | --- | --- |
| LSR1 R1 | 45,295,795 | 94.5 | 13,308 |
| LSR1 R2 | 45,162,410 | 93.2 | 13,276 |
| LSR2 R1 | 40,063,548 | 94.5 | 13,238 |
| LSR2 R2 | 40,013,314 | 93.1 | 13,287 |
| LSR3 R1 | 35,718,650 | 93.9 | 13,034 |
| LSR3 R2 | 35,643,040 | 92.7 | 13,068 |
| LSR4 R1 | 38,665,456 | 94.6 | 12,837 |
| LSR4 R2 | 38,554,412 | 93.2 | 12,915 |
| LSR5 R1 | 35,393,410 | 95.0 | 13,054 |
| LSR5 R2 | 35,269,826 | 93.6 | 13,062 |
| LSR6 R1 | 34,819,314 | 93.7 | 13,186 |
| LSR6 R2 | 34,701,966 | 92.4 | 13,251 |
| HSR1 R1 | 34,765,022 | 93.9 | 12,821 |
| HSR1 R2 | 34,664,912 | 92.5 | 12,878 |
| HSR2 R1 | 41,036,578 | 94.4 | 12,616 |
| HSR2 R2 | 40,914,866 | 92.9 | 12,859 |
| HSR3 R1 | 34,044,032 | 94.6 | 12,889 |
| HSR3 R2 | 33,927,046 | 93.3 | 12,888 |
| HSR4 R1 | 38,231,108 | 94.5 | 12,774 |
| HSR4 R2 | 38,132,004 | 93.1 | 12,758 |
| HSR5 R1 | 38,983,920 | 94.6 | 12,898 |
| HSR5 R2 | 38,877,926 | 93.4 | 12,880 |
| HSR6 R1 | 45,942,492 | 94.0 | 12,447 |
| HSR6 R2 | 45,800,282 | 92.5 | 12,427 |
