# Supplementary material for: Expression QTL (eQTLs) Analyses Reveal Candidate Genes Associated With Fruit Flesh Softening Rate in Peach [Prunus persica (L.) Batsch]
Source: Front Plant Sci. 2019 Dec 3;10:1581. doi: 10.3389/fpls.2019.01581 (PMC6901599; doi:10.3389/fpls.2019.01581)
Supplement: Supplementary file 9 [file Table_5.docx]

**Supplementary Table 5.** List of 133 differentially expressed (DE) genes that showed an eQTL peak over 5 and co-localized with the conventional QTL located on chromosome 4.

| **Gene _ID** | **Pos (cM)** | **LOD score** | **Putative function** | **Cis/Trans effect** |
| --- | --- | --- | --- | --- |
| Prupe.2G195100 | 36.5 | 8.63 | Rho GTPase activating protein with PAK-box/P21-Rho-binding domain | Trans |
| Prupe.2G180700 | 36.5 | 8.16 | Unknown Protein Function | Trans |
| Prupe.4G101400 | 36.5 | 7.52 | Hydroxyproline-rich glycoprotein family protein | Cis |
| Prupe.1G305400 | 36.0 | 7.41 | ROTUNDIFOLIA like 17 | Trans |
| Prupe.6G064000 | 36.0 | 7.40 | Protein of unknown function (DUF674) | Trans |
| Prupe.1G310900 | 36.0 | 7.11 | B-box type zinc finger protein with CCT domain | Trans |
| Prupe.3G314600 | 36.0 | 7.07 | nudix hydrolase homolog 12 | Trans |
| Prupe.7G105200 | 36.0 | 6.95 | HSP20-like chaperones superfamily protein | Trans |
| Prupe.1G472500 | 36.0 | 6.94 | HCO3- transporter family | Trans |
| Prupe.5G086900 | 36.5 | 6.93 | PGR5-LIKE A | Trans |
| Prupe.1G572200 | 36.0 | 6.91 | potassium transport 2/3 | Trans |
| Prupe.7G134600 | 41.0 | 6.90 | Unknown Protein Function | Trans |
| Prupe.8G081400 | 36.0 | 6.85 | SAUR-like auxin-responsive protein family | Trans |
| Prupe.3G214400 | 36.5 | 6.84 | cellulose synthase-like B4 | Trans |
| Prupe.1G215900 | 36.0 | 6.71 | P-loop containing nucleoside triphosphate hydrolases superfamily protein | Trans |
| Prupe.6G342800 | 36.5 | 6.71 | GDP-D-mannose 3\',5\'-epimerase | Trans |
| **Gene _ID** | **Pos (cM)** | **LOD score** | **Putative function** | **Cis/Trans effect** |
| Prupe.5G191200 | 36.5 | 6.62 | TEOSINTE BRANCHED, cycloidea and PCF (TCP) 14 | Trans |
| Prupe.7G235900 | 36.5 | 6.59 | Homeodomain-like superfamily protein | Trans |
| Prupe.5G059700 | 36.5 | 6.57 | FAD/NAD(P)-binding oxidoreductase family protein | Trans |
| Prupe.1G414800 | 35.0 | 6.52 | Protein phosphatase 2C family protein | Trans |
| Prupe.6G001500 | 36.5 | 6.47 | RING/U-box superfamily protein | Trans |
| Prupe.5G169700 | 36.0 | 6.43 | Leucine-rich receptor-like protein kinase family protein | Trans |
| Prupe.3G153300 | 36.5 | 6.38 | Unknown Protein Function | Trans |
| Prupe.3G239500 | 36.5 | 6.37 | Zinc-finger domain of monoamine-oxidase A repressor R1 protein | Trans |
| Prupe.8G247900 | 36.5 | 6.35 | resistance to phytophthora 1 | Trans |
| Prupe.7G196500 | 36.0 | 6.34 | strictosidine synthase-like 4 | Trans |
| Prupe.4G076400 | 36.5 | 6.28 | Unknown Protein Function | Cis |
| Prupe.3G179400 | 36.5 | 6.23 | photosystem II subunit P-1 | Trans |
| Prupe.1G472800 | 36.5 | 6.23 | Protein of unknown function, DUF642 | Trans |
| Prupe.8G012600 | 36.0 | 6.21 | ACT domain repeat 3 | Trans |
| Prupe.5G099500 | 36.0 | 6.15 | beta-6 tubulin | Trans |
| Prupe.2G303200 | 36.5 | 6.13 | Late embryogenesis abundant (LEA) hydroxyproline-rich glycoprotein family | Trans |
| Prupe.6G150900 | 36.0 | 6.08 | aldehyde oxidase 4 | Trans |
| Prupe.3G251500 | 36.5 | 6.07 | Unknown Protein Function | Trans |
| Prupe.6G228300 | 36.0 | 6.05 | Protein kinase superfamily protein | Trans |
| **Gene _ID** | **Pos (cM)** | **LOD score** | **Putative function** | **Cis/Trans effect** |
| Prupe.5G156300 | 36.5 | 6.05 | Unknown Protein Function | Trans |
| Prupe.6G264700 | 36.5 | 6.01 | Bifunctional inhibitor/lipid-transfer protein/seed storage 2S albumin superfamily protein | Trans |
| Prupe.4G185700 | 34.7 | 6.01 | protochlorophyllide oxidoreductase A | Cis |
| Prupe.2G294400 | 36.0 | 5.96 | CAP160 protein | Trans |
| Prupe.8G216700 | 36.5 | 5.95 | HAD superfamily, subfamily IIIB acid phosphatase | Trans |
| Prupe.1G276700 | 36.5 | 5.93 | expansin A1 | Trans |
| Prupe.2G232000 | 36.5 | 5.93 | Histone superfamily protein | Trans |
| Prupe.1G555600 | 36.5 | 5.93 | alpha/beta-Hydrolases superfamily protein | Trans |
| Prupe.1G429700 | 37.0 | 5.86 | S-locus lectin protein kinase family protein | Trans |
| Prupe.1G271700 | 36.0 | 5.86 | basic helix-loop-helix (bHLH) DNA-binding superfamily protein | Trans |
| Prupe.8G079200 | 36.5 | 5.85 | SAUR-like auxin-responsive protein family | Trans |
| Prupe.6G147100 | 36.5 | 5.83 | MD-2-related lipid recognition domain-containing protein / ML domain-containing protein | Trans |
| Prupe.2G263300 | 36.5 | 5.83 | Unknown Protein Function | Trans |
| Prupe.2G222100 | 36.5 | 5.80 | Unknown Protein Function | Trans |
| Prupe.1G545200 | 36.5 | 5.79 | NB-ARC domain-containing disease resistance protein | Trans |
| Prupe.3G179800 | 36.0 | 5.78 | phytochrome interacting factor 4 | Trans |
| **Gene _ID** | **Pos (cM)** | **LOD score** | **Putative function** | **Cis/Trans effect** |
| Prupe.2G328200 | 36.5 | 5.77 | Plant protein of unknown function (DUF827) | Trans |
| Prupe.5G188300 | 36.5 | 5.76 | IQ-domain 31 | Trans |
| Prupe.3G162800 | 36.0 | 5.75 | Calcium-dependent lipid-binding (CaLB domain) family protein | Trans |
| Prupe.8G081300 | 36.0 | 5.73 | SAUR-like auxin-responsive protein family | Trans |
| Prupe.1G419700 | 36.5 | 5.72 | basic leucine-zipper 4 | Trans |
| Prupe.2G273500 | 41.0 | 5.72 | DERLIN-1 | Trans |
| Prupe.7G078000 | 41.0 | 5.71 | NB-ARC domain-containing disease resistance protein | Trans |
| Prupe.2G231900 | 36.5 | 5.67 | ACT domain-containing protein | Trans |
| Prupe.3G001400 | 41.0 | 5.66 | Unknown Protein Function | Trans |
| Prupe.3G312500 | 36.0 | 5.66 | Unknown Protein Function | Trans |
| Prupe.5G044900 | 36.5 | 5.65 | Unknown Protein Function | Trans |
| Prupe.4G054300 | 36.5 | 5.63 | Histone superfamily protein | Cis |
| Prupe.6G016200 | 36.5 | 5.60 | Histone superfamily protein | Trans |
| Prupe.6G287000 | 36.5 | 5.59 | PLAC8 family protein | Trans |
| Prupe.7G012500 | 35.0 | 5.57 | cationic amino acid transporter 2 | Trans |
| Prupe.5G044700 | 34.7 | 5.56 | Unknown Protein Function | Trans |
| Prupe.7G158400 | 36.5 | 5.53 | S-adenosyl-L-methionine-dependent methyltransferases superfamily protein | Trans |
| Prupe.7G169200 | 35.0 | 5.53 | ferredoxin-NADP(+)-oxidoreductase 1 | Trans |
| **Gene _ID** | **Pos (cM)** | **LOD score** | **Putative function** | **Cis/Trans effect** |
| Prupe.1G013900 | 36.5 | 5.51 | 2Fe-2S ferredoxin-like superfamily protein | Trans |
| Prupe.8G078700 | 36.5 | 5.49 | SAUR-like auxin-responsive protein family | Trans |
| Prupe.4G125700 | 36.5 | 5.49 | DNA glycosylase superfamily protein | Cis |
| Prupe.4G225800 | 36.0 | 5.49 | Unknown | Cis |
| Prupe.3G201000 | 36.0 | 5.49 | photosystem II light harvesting complex gene 2.2 | Trans |
| Prupe.7G172400 | 36.0 | 5.49 | Small GTP-binding protein | Trans |
| Prupe.5G236500 | 36.5 | 5.46 | potassium transporter 2 | Trans |
| Prupe.1G050900 | 36.5 | 5.42 | AWPM-19-like family protein | Trans |
| Prupe.1G325900 | 36.5 | 5.42 | nudix hydrolase homolog 25 | Trans |
| Prupe.3G055000 | 36.0 | 5.4 | myo-inositol oxygenase 1 | Trans |
| Prupe.1G527600 | 41.0 | 5.37 | sequence-specific DNA binding transcription factors;transcription regulators | Trans |
| Prupe.1G527700 | 36.0 | 5.36 | conserved peptide upstream open reading frame 37 | Trans |
| Prupe.1G064600 | 36.5 | 5.33 | Protein of unknown function (DUF581) | Trans |
| Prupe.4G201400 | 36.0 | 5.33 | C-terminal cysteine residue is changed to a serine 1 | Cis |
| Prupe.4G173700 | 36.0 | 5.32 | UDP-Glycosyltransferase superfamily protein | Cis |
| Prupe.8G081100 | 35.0 | 5.31 | SAUR-like auxin-responsive protein family | Trans |
| Prupe.2G271100 | 36.5 | 5.31 | cyclin p4;1 | Trans |
| Prupe.8G162500 | 36.5 | 5.31 | PLC-like phosphodiesterases superfamily protein | Trans |
| **Gene _ID** | **Pos (cM)** | **LOD score** | **Putative function** | **Cis/Trans effect** |
| Prupe.5G148500 | 36.5 | 5.30 | Heavy metal transport/detoxification superfamily protein | Trans |
| Prupe.6G231800 | 36.5 | 5.30 | Plant protein of unknown function (DUF869) | Trans |
| Prupe.4G200500 | 36.5 | 5.30 | NAD(P)-binding Rossmann-fold superfamily protein | Cis |
| Prupe.2G168300 | 36.5 | 5.30 | Unknown | Trans |
| Prupe.2G275500 | 36.5 | 5.29 | Leucine-rich repeat protein kinase family protein | Trans |
| Prupe.3G036700 | 36.0 | 5.29 | 2-oxoglutarate-dependent dioxygenase family protein | Trans |
| Prupe.2G091600 | 36.5 | 5.28 | lactate/malate dehydrogenase family protein | Trans |
| Prupe.8G254300 | 36.5 | 5.28 | Thioredoxin superfamily protein | Trans |
| Prupe.8G256300 | 36.5 | 5.27 | WEE1 kinase homolog | Trans |
| Prupe.5G101200 | 36.5 | 5.26 | Unknown Protein Function | Trans |
| Prupe.7G197300 | 41.0 | 5.26 | syntaxin of plants 121 | Trans |
| Prupe.5G188200 | 36.5 | 5.26 | Unknown Protein Function | Trans |
| Prupe.6G009900 | 36.5 | 5.24 | Bifunctional inhibitor/lipid-transfer protein/seed storage 2S albumin superfamily protein | Trans |
| Prupe.4G165300 | 36.5 | 5.24 | Protein of unknown function (DUF640) | Cis |
| Prupe.6G273000 | 36.0 | 5.23 | Transmembrane amino acid transporter family protein | Trans |
| Prupe.2G238800 | 36.0 | 5.22 | rubisco activase | Trans |
| **Gene _ID** | **Pos (cM)** | **LOD score** | **Putative function** | **Cis/Trans effect** |
| Prupe.4G144300 | 36.5 | 5.22 | Translation initiation factor 2, small GTP-binding protein | Cis |
| Prupe.6G264800 | 36.5 | 5.22 | RHO guanyl-nucleotide exchange factor 7 | Trans |
| Prupe.5G027800 | 41.0 | 5.22 | xyloglucan endotransglucosylase/hydrolase 30 | Trans |
| Prupe.5G178300 | 41.0 | 5.22 | Calmodulin binding protein-like | Trans |
| Prupe.7G063100 | 36.5 | 5.21 | Leucine-rich repeat protein kinase family protein | Trans |
| Prupe.4G271200 | 36.5 | 5.18 | photosystem I reaction center subunit PSI-N, chloroplast, putative / PSI-N, putative (PSAN) | Cis |
| Prupe.4G196000 | 35.0 | 5.18 | receptor kinase 3 | Cis |
| Prupe.1G342300 | 35.0 | 5.18 | P-loop containing nucleoside triphosphate hydrolases superfamily protein | Trans |
| Prupe.8G076200 | 43.0 | 5.17 | Octicosapeptide/Phox/Bem1p family protein | Trans |
| Prupe.1G491000 | 36.0 | 5.17 | amino acid permease 2 | Trans |
| Prupe.2G036000 | 36.0 | 5.16 | PHD finger family protein / bromo-adjacent homology (BAH) domain-containing protein | Trans |
| Prupe.2G147700 | 36.5 | 5.15 | FASCICLIN-like arabinogalactan protein 8 | Trans |
| Prupe.2G106400 | 36.0 | 5.14 | Unknown Protein Function | Trans |
| Prupe.6G267400 | 36.0 | 5.13 | Protein kinase protein with tetratricopeptide repeat domain | Trans |
| Prupe.5G217700 | 36.5 | 5.13 | Duplicated homeodomain-like superfamily protein | Trans |
| **Gene _ID** | **Pos (cM)** | **LOD score** | **Putative function** | **Cis/Trans effect** |
| Prupe.6G229300 | 36.5 | 5.12 | end binding protein 1C | Trans |
| Prupe.1G475100 | 36.0 | 5.10 | BURP domain-containing protein | Trans |
| Prupe.2G232200 | 36.5 | 5.08 | Histone superfamily protein | Trans |
| Prupe.1G446400 | 34.7 | 5.06 | Polyketide cyclase/dehydrase and lipid transport superfamily protein | Trans |
| Prupe.2G308300 | 36.0 | 5.06 | Haloacid dehalogenase-like hydrolase (HAD) superfamily protein | Trans |
| Prupe.8G097500 | 41.0 | 5.05 | Protein kinase superfamily protein | Trans |
| Prupe.1G216600 | 36.5 | 5.05 | Protein kinase superfamily protein | Trans |
| Prupe.1G388400 | 37.0 | 5.05 | Pyridoxal phosphate (PLP)-dependent transferases superfamily protein | Trans |
| Prupe.3G285700 | 36.5 | 5.03 | like SEX4 1 | Trans |
| Prupe.3G242700 | 36.5 | 5.03 | zinc finger (C3HC4-type RING finger) family protein / BRCT domain-containing protein | Trans |
| Prupe.7G158900 | 36.5 | 5.02 | cytochrome BC1 synthesis | Trans |
| Prupe.3G153100 | 36.5 | 5.02 | Unknown Protein Function | Trans |
| Prupe.5G024500 | 39.0 | 5.02 | membrane protein, putative | Trans |
| Prupe.1G386700 | 45.6 | 5.01 | Protein kinase protein with adenine nucleotide alpha hydrolases-like domain | Trans |
| Prupe.1G450700 | 36.5 | 5.00 | ARM repeat superfamily protein | Trans |
