# Supplementary material for: Expression QTL (eQTLs) Analyses Reveal Candidate Genes Associated With Fruit Flesh Softening Rate in Peach [Prunus persica (L.) Batsch]
Source: Front Plant Sci. 2019 Dec 3;10:1581. doi: 10.3389/fpls.2019.01581 (PMC6901599; doi:10.3389/fpls.2019.01581)
Supplement: Supplementary file 10 [file Table_6.docx]

**Supplementary Table 6**. Primers designed for the analysis of the expression level of the six candidate genes, based on real-time qPCR.

| **Gene_ID** | **Gene name** | **Primer** | **Sequence (5'->3')** | **Tm** | **GC%** |
| --- | --- | --- | --- | --- | --- |
| Prupe.1G332600 | RNA-dependent RNA polymerase | PF | CTGGAGAAGGGACTGTTCATGC | 60.94 | 54.55 |
|  |  | PR | GTACCACAGGCTTCGAGCATCGGC | 67.76 | 62.50 |
| Prupe.1G460100 | Unknown Protein Function | PF | CAGGGAAGGAGCTGAAGAAGAG | 60.09 | 54.55 |
|  |  | PR | GTAGGCCTCAGATAGTTTGGGAC | 60.18 | 52.17 |
| Prupe.3G255800 | Cyclin-like family protein | PF | CCGACCCTATAACGGACAATGGTGT | 64.37 | 52.00 |
|  |  | PR | CGTGGCAACGAGTGCTAGCCCG | 68.27 | 68.18 |
| Prupe.5G130800 | Unknown Protein Function | PF | GGCAAGGCCAAATCTGCTTTG | 60.94 | 52.38 |
|  |  | PR | GGATGAGGAGCAATAGCAAGGC | 61.39 | 54.55 |
| Prupe.7G216300 | Inositol oxygenase 4 | PF | CGAACCACATCAATCAAACATTCG | 59.45 | 41.67 |
|  |  | PR | CTGATGGCTTCAGCTGTTTG | 57.37 | 50.00 |
| Prupe.8G079500 | Indole-3-acetic acid-induced protein ARG7 | PF | GCAGTGTATGTTGGGGAGAGCCAG | 65.28 | 58.33 |
|  |  | PR | GTCTTCACTGCAGGGGATGGTG | 63.15 | 59.09 |

PF= Primer forward, PR= Primer reverse.
